# Recognizing puzzling PD1 + infiltrates in marginal zone lymphoma by integrating clonal and mutational findings: pitfalls in both nodal and transformed splenic cases

**DOI:** 10.1186/s13000-023-01422-9

**Published:** 2023-12-11

**Authors:** Jili Deng, Youjun Cao, Xinting Diao, Meng Wu, Xianghong Li, Yunfei Shi

**Affiliations:** Key Laboratory of Carcinogenesis and Translational Research (Ministry of Education/Beijing), Department of Pathology, Peking University Cancer Hospital & Institute, Beijing 100142, China

**Keywords:** Clonality, Genetic mutation, Marginal zone lymphoma, PD1, Pitfall, T cell, Case report

## Abstract

**Background:**

A marked increase in PD1-positive TFH cells in nodal MZL cases (NMZL) was reported previously and could prompt suspicion for a diagnosis of peripheral T-cell lymphoma (PTCL), especially angioimmunoblastic T-cell lymphoma (AITL).

**Case presentation:**

To demonstrate that the pitfall might exist not only in NMZL but also in transformed splenic MZL (tSMZL), two NMZL cases (70 y/o female with enlarged left cervical lymph node and 75 y/o male with generalized lymphadenopathy) and one case of tSMZL (47 y/o male with nodal and extranodal involvement) with obvious PD1-positive T-cell hyperplasia were described here. Although all their initial diagnoses were prompted to be AITL, they were comprehensively characterized by clinical features, morphologic, immunophenotypic, clonality, and targeted exosome sequencing (TES) findings. Case 1 and Case 2 were NMZL with increased PD1 + T cells in the “peripheral pattern” or “mixed peripheral and central pattern”, and Case 3 was SMZL with abundant PD1-positive T cells in the “nodular pattern” that transformed to tSMZL (DLBCL) with PD1-positive T cells distributed in the “diffuse pattern.” In addition to the monoclonal IG rearrangement and polyclonal TCR rearrangement results, TES demonstrated enriched and recurrent mutations in MZLs and failed to find aberrations described in AITL- or TFH-derived lymphomas.

**Conclusions:**

It is important to realize that this pitfall can also occur in more diagnostically difficult tSMZL cases; the integration of histopathology with clonality and mutation studies is also highlighted.

**Supplementary Information:**

The online version contains supplementary material available at 10.1186/s13000-023-01422-9.

## Introduction

There are three subtypes of marginal zone lymphoma (MZL): nodal MZL (NMZL), extranodal MZL, and splenic MZL (SMZL) [1]. In some MZL cases, an increase in PD1-positive cells could lead to misdiagnosis of peripheral T-cell lymphoma (PTCL), especially angioimmunoblastic T-cell lymphoma (AITL) [2].

Here, we described PD1-positive T-cell hyperplasia in two NMZL cases and a rarely described case of transformed SMZL (tSMZL), which were all proposed to be AITL or PTCL before clonality and mutational analysis. Thus, the pitfall can also be seen in tSMZL.

## Case 1

### Case presentation

A 70-year-old (y/o) Chinese woman had an enlarged left cervical lymph node in May 2022, and the node shrunk slightly after anti-inflammatory treatment. Physical examination found that multiple lymph nodes were enlarged, accompanied by weight loss of 5 kg, but with no fever or night sweats. In July 2022, a left inguinal excisional lymph node biopsy suggested atypical T-cell hyperplasia, highly suggestive of AITL; however, further clonality analysis and NGS revealed the final diagnosis to be “NMZL accompanied by atypical PD1 + cell proliferation.” As the patient had no physical discomfort, she was follow-up was recommended.

### Pathological findings

**Fig. 1:**
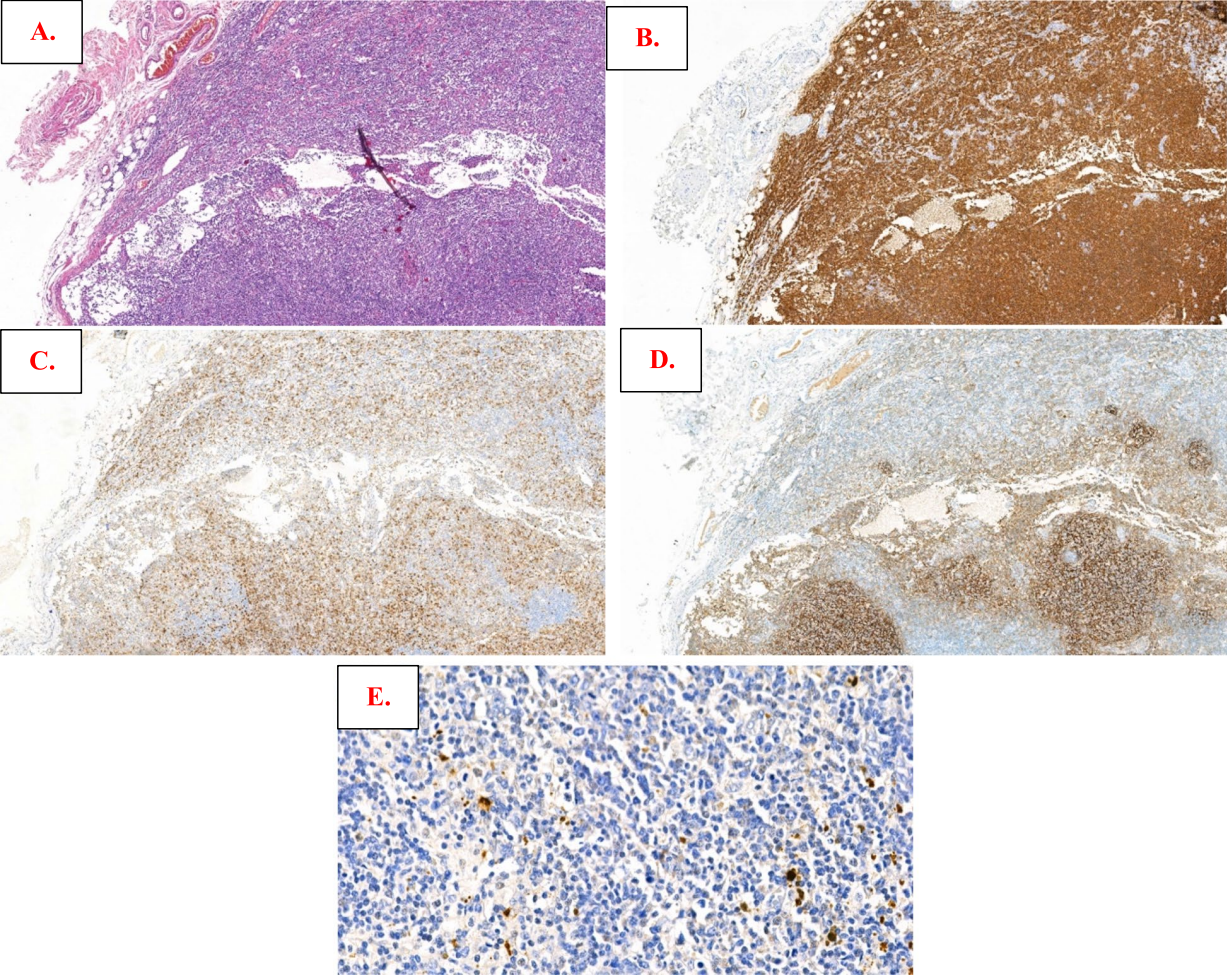
Morphologic and immunophenotypic findings of lymphoma in Case 1. The neoplastic lymphoid cells infiltrated diffusely with vague nodules in (**A)**; they were small to medium in size and by immunostaining; they were positive for CD20 in (**B**); quantities of perifollicular T-cell infiltrates that were PD1-positive were seen in (**C**); the follicular dendritic cell meshwork mainly confined to follicles were also shown by CD21 in (**D**); by in situ hybridization with Epstein–Barr virus-encoded small RNAs, rare small positive cells (**E**) were also noted

There were abundant neoplastic lymphoid cells (Fig. 1A), most of which were medium in size and were of B-cell lineage (decorated by CD20, Fig. 1B). There were some clusters of dense perifollicular infiltrates with clear cytoplasm, and they were composed of PD1-positive T cells (Fig. 1C) shown by CD21. The expanded follicular dendritic cell (FDC) meshwork was confined to follicles (Fig. 1D). In addition, rarely disseminated small EBER + cells could be seen (Fig. 1E, see more extensive morphologic and immunophenotypic features in Figure S1), raising the possibility of angioimmunoblastic T-cell lymphoma (AITL, pattern I). Clonality analysis was ordered, and clonal IG gene rearrangements and polyclonal TCR gene rearrangements were found (Figure S2). Targeted exome sequencing (TES) detected mutations in *KMT2D (*1 deletion mutation, DEL and 1 frameshift mutation, FS), *NOTCH2* (1 FS), *TBL1XR1* (1 MS and 1 stop-gain mutation, SG)*, TNFAIP3* (1FS) and *TNFRSF14* (1 DEL), and AITL recurrent aberrations (such as *RHOA*, *IDH2*, *DNMT3A* and *TET2*) were not observed (details in Table S1). Thus, it was in concordance with “NMZL with increased peripheral PD1 + T cells.”

## Case 2

### Case presentation

A 75 y/o Chinese man sought treatment for axillary, inguinal, and cervical lymphadenopathy in October 2021. He was hospitalized with fever in the local hospital, anti-infection treatment was ineffective, and the diagnosis was unclear. Laboratory values were as follows: hemoglobin, 12.0 g/dL; WBCs, 4.5 × 103/μL; and platelets, 84.0 × 103/μL. Whole-body [18F]-2-fluoro-2-deoxy-D-glucose (FDG)-positron emission tomography/computed tomography (PET/CT) demonstrated systemic FDG–avid enlarged lymph nodes (bilateral neck, bilateral clavicle area, bilateral armpit, bilateral internal mammary area, bilateral diaphragmatic area, abdominal and pelvic cavity, retroperitoneum, bilateral iliac vessels, bilateral inguinal area), with a maximum standardized uptake value (SUVmax) of 3.1 in the retroperitoneal lymph node. Increased accumulation of FDG in the spleen (SUVmax 6.7) and bone marrow (SUVmax 3.9) was also noted. A subsequent biopsy of a right groin lymph node was performed and was interpreted as being in concordance with AITL, but clonality and TES were suggested. The final diagnosis was “Nodal Marginal Zone Lymphoma with Robust PD1-positive T-Cell Hyperplasia” after clonality and mutational analysis. Strict follow-up observation was adopted.

### Pathological findings

**Fig. 2:**
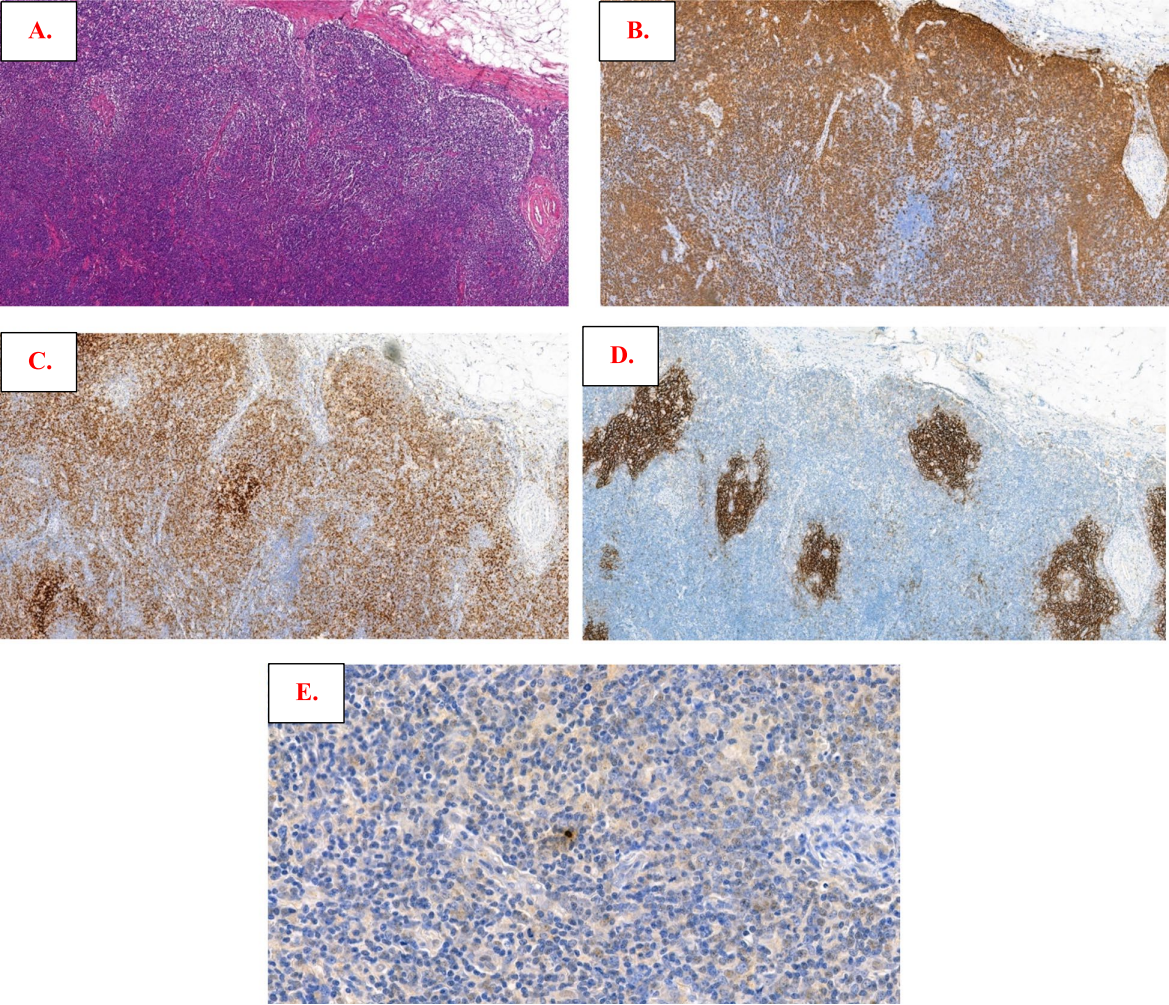
Morphologic and immunophenotypic findings of lymphoma in Case 2. Follicles were regressed, and the B-cell population was reduced (**A**) as also shown by CD20 immunostaining (**B**); extensive accumulation of PD-1-positive cells was observed in both centralized and peripheralized patterns around the follicles (**C**); an irregularly enveloped follicular dendritic cell meshwork is revealed by CD21 (**D**); rare small positive lymphoid cells were also noted by in situ hybridization with Epstein–Barr virus-encoded small RNAs (**E**)

With regressed follicles and a reduced B-cell population (also shown by CD20, Fig. 2A and B), extensive accumulation of PD-1-positive cells can be seen by IHC, not only in a centralized intrafollicular pattern but also in a peripheralized pattern (Fig. 2C). The exaggerated PD1-positive cells enveloped and surrounded regressed FDC meshworks as shown by CD21 (Fig. 2D). In addition, EBER was also positive in rare small lymphoid cells (Fig. 2E). More extensive morphologic and immunophenotypic features can be seen in Figure S3. The extensive accumulation of T cells and reduced B cells might suggest the development of angioimmunoblastic T-cell lymphoma (AITL, pattern II). Clonality analysis report indicated monoclonal IG gene rearrangements and polyclonal TCR gene rearrangements (Figure S4), and TES indicated mutations in *EP300*(1MS), *KMT2C*(1MS) and *KMT2D*(3FS) and no AITL associated aberrations (Table S1). NMZL with PD1-positive T-cell expansion (mixed peripheral and central patterns) was observed.

## Case 3

### Case presentation

**Fig. 3:**
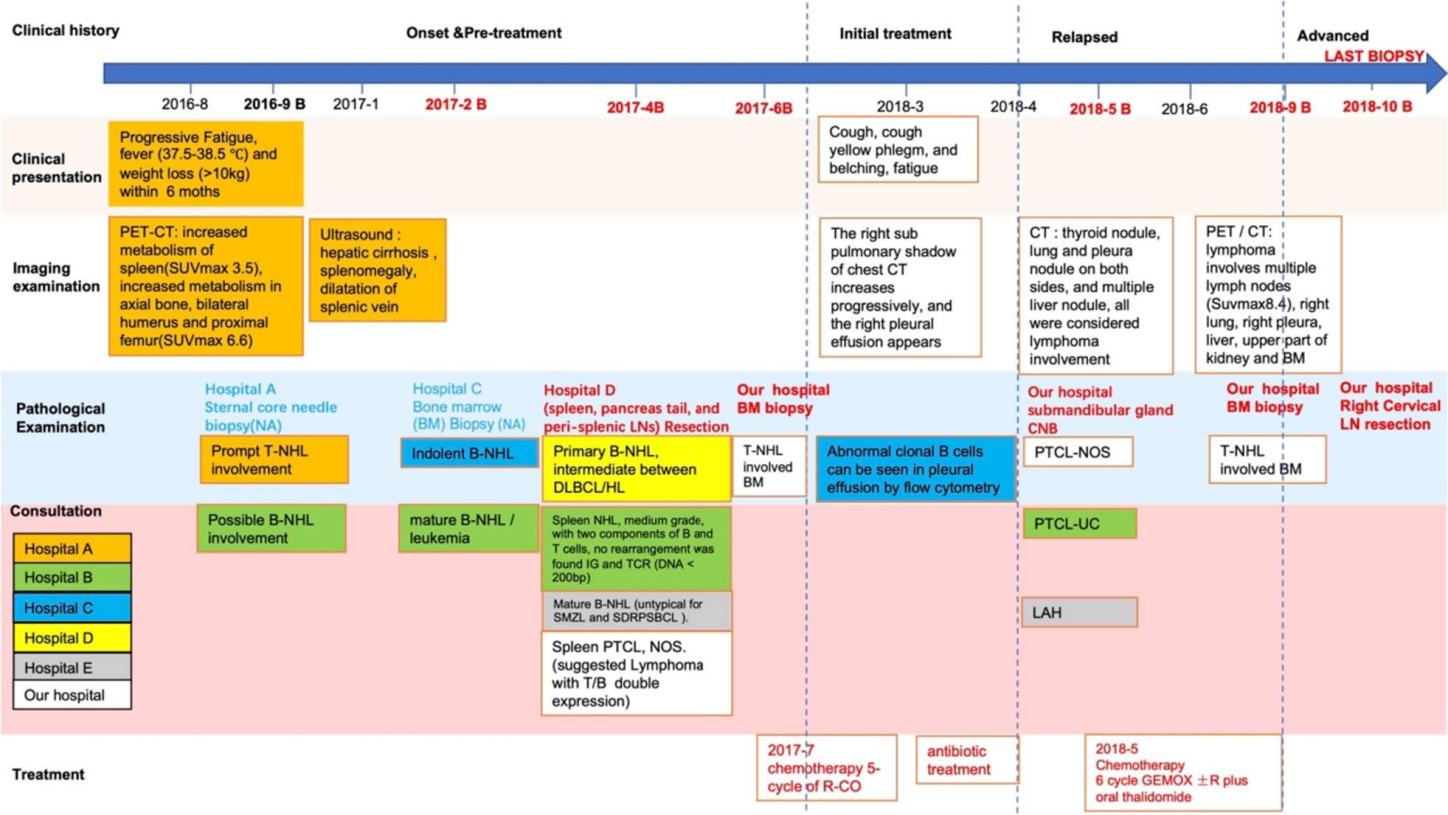
Clinical history of a transformed splenic marginal zone lymphoma (Case 3)

The patient was a 47-year-old Chinese male who suffered pancytopenia, progressive fatigue, fever (37.5–38.5 °C) and weight loss (> 10 kg) since August 2016. He also had a previous history of chronic hepatitis B. PET/CT (2016–8) showed an enlarged spleen with an SUVmax of 3.5 and diffusely high FDG uptake in the bone marrow (SUV max 6.6). Then he underwent bone marrow biopsy twice (not available), which was suspected to be indolent B-cell lymphoma. To confirm the diagnosis and pathological subtype, he underwent splenectomy with pancreatic tail and perisplenic LNs in April 2017, and controversially, the pathological diagnosis was either B-NHL (from indolent to aggressive), T-NHL, composite T-NHL & B-NHL, or gray zone lymphoma (CHL/DLBCL) with pathology consultation across 6 hospitals. From July 2017 to December 2017, he received 5 cycles of R-CO (rituximab + cyclophosphamide + vindesine) and 3 cycles of R-CCO (rituximab + cyclophosphamide + vindesine + doxorubicin liposome); he achieved PR at the end of treatment assessment (residual disease in bone marrow). Unfortunately, 5 months after his first-line therapy, PET/CT showed involvement in the right salivary gland, thyroid, lung, right pleura, and liver. After relapse proved by core needle biopsy (CNB) from the salivary gland in May 2018, lymphoma with a pathological diagnosis suggestive of PTCL was diagnosed, and he received 6 cycles of GEMOX ± R plus thalidomide. However, his disease progressed again on October 2018. His cervical LN was biopsied again. However, the pathological characteristics still supported different opinions regarding the diagnosis of “PTCL with TFH Immunol phenotype,” “T-cell and histocyte-rich DLBCL” or “aggressive B-cell lymphoma.” From October 2018 to November 2018, he received EPOCH × 2 cycles; however, after a short interval of partial remission, the patient died in December 2018 (Fig. 3).

### Pathological findings

**Fig. 4:**
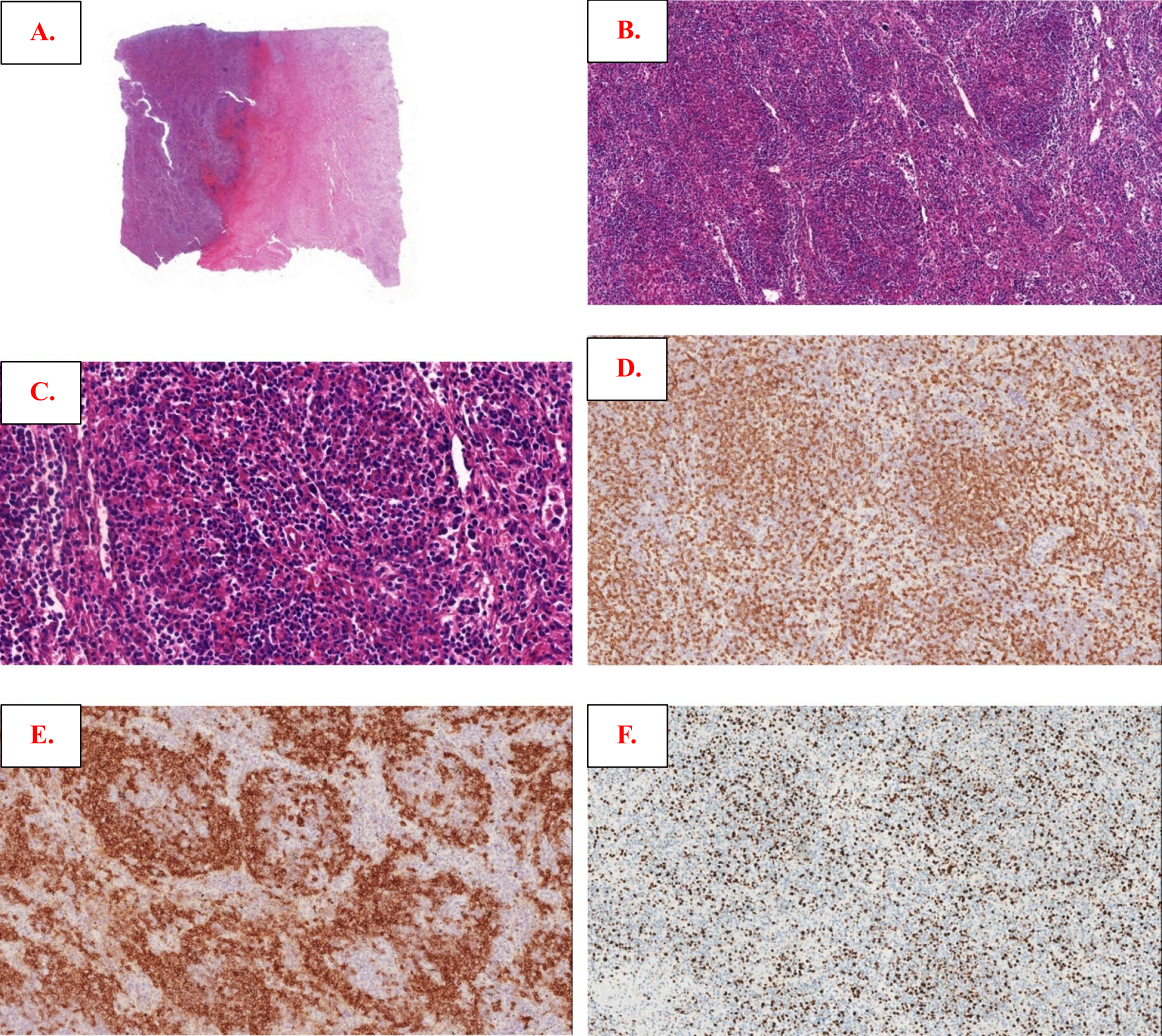
Morphologic and immunophenotypic findings of lymphoma from April 2017 splenectomy in Case 3. Spleen structure replaced neoplastic lymphoid cells with necrosis in (**A**); nodular growth pattern can be seen easily in (**B**), comprising small- to medium-sized cells in (**C**); by immunostaining, cells in the center region of nodules were CD3 positive (**D**), cells in the peripheral region of the nodules were CD20 positive (**E**), and the Ki67 index was approximately 35% in the center and 10% in the periphery of the nodules (**F**)

**Fig. 5:**
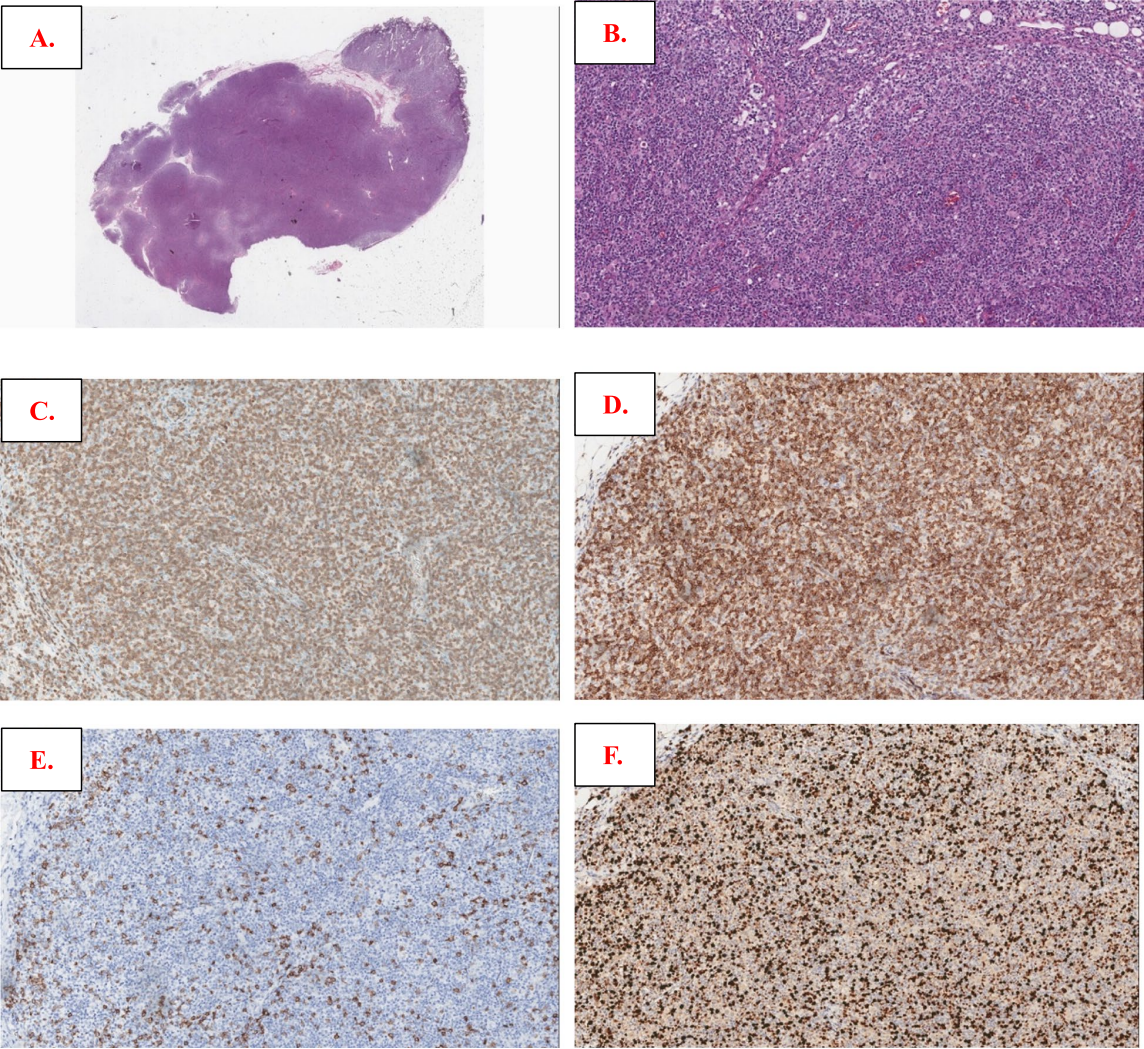
Morphologic and immunophenotypic findings from the October 2018 cervical lymph node biopsy in Case 3. Atypical lymphocytes infiltrated more diffusely in (**A**), and most were small to medium in size with clear to pale cytoplasm (**B**); most smaller cells were positive for CD3 (**C**) and PD1 (**D**), while scattered CD19-positive (**E**) and CD22-positive (not shown here) larger immunoblastic-like B cells were also mentioned, with a Ki67 index of nearly 50% (**F**)

Regarding splenectomy in April 2017, the normal architecture was completely replaced by nodules composed of small- to medium-sized lymphoid cells, with substantial necrosis on one side. The cells in the center of the nodules were positive for CD3 and CD5, while the cells in the periphery of the nodules were positive for CD20 and PAX5, with a Ki-67 index of approximately 35% in the center and nearly 10% peripherally (Fig. 4A-E). Similar lymphocyte nodules occupying most of the intertrabecular space were observed in the bone marrow (BM) biopsy section in June 2017 (Figure S5A-D). Regarding the submandibular gland core biopsy in May 2018, most cells were smaller and were CD3 + T cells without a nodular pattern, and there were scattered CD20-positive atypical immunoblastic-like lymphocytes, with an increased Ki67 of approximately 50% (Figure S6A-E). There were still fewer atypical lymphocyte nodules in the spleen with the subsequent September 2018 BM biopsy as mentioned previously. The CD3-positive cells were still located in the center (Figure S7A-B) of the nodules, but the atypical B cells turned CD20 negative (not shown), probably because of the usage of rituximab. However, PD1 testing was not performed among all specimens mentioned above. For the last LN resection biopsied in October 2018, there were diffuse infiltrating atypical lymphocytes; most lymphocytes were small to medium in size with clear to pale cytoplasm and were diffusely positive for CD3 and PD1. There were also some scattered CD22&CD19 positive large immunoblast-like B cells, and CD20 was negative (not shown), with an increased Ki67 index of nearly 50% (Fig. 5A-F). All the samples from Case 3 were negative for EBER. Splenectomy specimens in April 2017 were performed in another laboratory and indicated monoclonal results for IG rearrangements and polyclonal results for TCR gene rearrangements (including IGH-A tube, IGH-B tube and IGK-B tube, details not available). Clonal IG gene rearrangements and polyclonal TCR gene rearrangements were present (see Figure S8) from the resected lymph node in October 2018. The initial differential diagnosis was controversial at that time and included “PTCL with TFH immunophenotype,” “T-cell and histocyte-rich DLBCL” and “aggressive B-cell lymphoma.” The TES for LN samples in October 2018 included *CD58*(1MS), *KMT2D*(1MS) and *TNFAIP3*(1FS, Table S1), without AITL-associated genetic aberrations. After TES and recognition of these puzzling T-cell infiltrates, the diagnosis of “transformed splenic MZL with robust PD1-positive T-cell hyperplasia” was convincingly reached.

All the pathological and molecular findings above are summarized in Table 1.

**Table 1:** Histological and molecular of MZL (or transformed MZL) with PD1-positive T-Cell expansions

	Case 1	Case 2	Case 3 (transformed)
Age (years)	70	75	47
Sex	Woman	Man	Man
Biopsy Location	left cervical lymph node	right groin lymph node	right cervical LN
B cells			
CD20	+	+	-
PAX5	+	+	-
CD19	NA	NA	+
CD22	NA	NA	+
CD10	-	-	-
BCL6	-	-	-
CD30	-	-	+
B cell size/Pattern	Small-medium /Diffuse	Small-medium/Nodular and disseminated	Medium-Large /Scattered
T cells			
CD3	+	+	+
CD4	+	+	+
CD8	subset +	subset +	subset +
CD2	+	+	+
CD5	+	+	+
CD7	+	+	+
PD1	+	+	+
CD10	Minor subset +	-	-
BCL6	+	subset +	Minor subset +
CXCL13	NA	+	Minor subset +
PD-1 positive T-cell localization	Reactive-like Pattern	Follicular Pattern peripheral and central mixed	Diffuse Pattern
CD21 + FDC cells	follicular	extrafollicular	Absent
EBER1/2	Rare	Rare	Negative
IG rearrangement	Monoclonal	Monoclonal	Monoclonal
TCR rearrangement	Polyclonal	Polyclonal	Polyclonal

## Discussion

In our study, in two cases of NMZL with increased PD1-positive T cells, IHC showed that the expanded FDC meshwork was restricted within lymphoid follicles in Case 1, and the meshwork was found with irregular contour in Case 2, resembling AITL with “pattern I” and “pattern II” [3]. In both cases, rare EBER-positive cells were in concordance with the common findings in AITL [4]. Finally, although predominant monotypic B-cell proliferation was also observed, this phenomenon might occur in AITL and other PTCLs, complicating the differentiation of MZL and AITL [3].

The diagnosis of MZL with a marked infiltrate of PD1-positive T cells was supported by monoclonal IG gene rearrangements and polyclonal TCR gene rearrangements. Targeted sequencing results showed recurrent mutated genes in MZL [5], but no genetic aberrations indicating TFH origin lymphomas were found [6–9]. Thus, in conjunction with morphological plus IHC and TCR/IG clonality studies, our TES studies confirmed the diagnosis of MZL.

Case 3 was diagnosed as T-cell non-Hodgkin lymphoma (T-NHL) and indolent B-NHL by 1st and 2nd BM biopsy, but prominent T-cell centralized nodules were seen in subsequent splenectomy specimens, so controversies of pathological diagnosis existed among different consultation centers, either B-NHL or PTCL-NOS. SMZL distributed in a pattern of T-cell-rich nodules was seldom mentioned previously [10, 11]. However, as considerable T-cell involvement was seen in the BM biopsy, irrespective of monoclonal IG/polyclonal TCR rearrangement in the splenectomy specimen, the tumor was more likely to be diagnosed as PTCL in our center. It was still considered to be PTCL in the atypical submandibular gland biopsy; enlarged B cells were ignored and repeated clonality studies were not ordered; the consideration of PTCL was reinforced by the T-cell infiltrates in subsequent BM sections. However, by integrating morphological, immunophenotypic and molecular studies, especially TES results, we found that it was a transformed splenic marginal zone lymphoma with prominent PD1-positive T cells.

PD1 + TFH cells have been reported in primary cutaneous MZL and represent an organoid immune response against cutaneous antigen stimuli [12]. However, much remains to be understood regarding the biological mechanisms and importance. In addition, exaggerated infiltration of PD1-positive T cells in MZL could still raise the possibility of immunotherapeutic modulation using immune checkpoint inhibitors.

## Conclusion

Realization and recognition of the existence of these puzzling PD-1 + T-cell infiltrates in NMZL and tSMZL is crucial. Integrating the utility of clonality and mutational analysis is recommended.

### Supplementary Information

**Additional file 1.** Supplementary Materials. Methods. Histology and Immunohistochemistry. Cases were collected from the Peking University Cancer Hospital and reviewed by an expert panel (YF.S., YM.L., and XH.L.), with a consensus diagnosis of MZL or tSMZL. The study was approved by the ethical committee of the institution and followed the 1964 Helsinki Declaration. Hematoxylin and eosin (H&E) and immunohistochemistry-stained slides from each case were evaluated. Immunohistochemistry was performed on FFPE sections on a Ventana Benchmark automated immunostainer using UltraView detection kits. The panel of antibodies can be seen in Table S2. The interpretation of the PD1 staining pattern included both the predominant location of the PD1-positive cells (follicular or extrafollicular) and was considered “normal” if most PD-1-positive cells were confined to intrafollicular areas and concentrated in the light zone. In situ hybridization to detect EBV-encoded RNA. EBV status was determined by in situ hybridization (ISH) to detect EBV-encoded RNA 1 and 2 (EBER1/2s) using peroxidase-labeled probes (ISH-7001UM, Beijing Zhongshan Golden Bridge Biotechnology). Tissue from a known EBV-positive nasopharyngeal carcinoma was used as a positive control. The EBV status was considered positive if at least one definitive cell expressed EBER. All H&E, IHC and ISH slides were independently and dual assessed. Immunoglobulin Gene and T-Cell Receptor Gene Rearrangement Studies. DNA was extracted from FFPE tissue sections using a QIAGEN QIAamp DNA FFPE Tissue Kit according to the manufacturer’s protocol (QIAGEN, Germantown, MD). Polymerase chain reaction (PCR) for immunoglobulin gene (IGH and IGK loci) and T-cell receptor (TCR locus) rearrangements was performed using commercially available BIOMED-2 multiplex PCR kits (Righton Gene, Shanghai). PCR products were separated by capillary electrophoresis and subjected to GeneScan analysis for confirmation of the monoclonal character of the IG or TCR gene rearrangements on an ABI 9700 Genetic Analyzer (Applied Biosystems, Foster City, CA), and electropherograms were analyzed using GeneMapper software, version 4.0. Targeted exome sequencing (TES) and sequence data analysis. Genomic DNA (gDNA) extraction from FFPE tissues, library preparation, and target gene enrichment were performed according to the manufacturer’s protocol. The gDNA libraries were subjected to high-throughput sequencing with 150-bp paired-end reads on the NovaSeq 60,000 Sequencing System (Illumina, San Diego, CA) supported by a commercial vendor (Geneplus-Beijing, China). The average sequencing depth of tissues was ~500×. Sequence reads were aligned using BWA version 0.5.9 (Broad Institute). Single nucleotide variants (SNVs) were called using MuTect (version 1.1.4) and NChot. Small insertions and deletions (Indels) were determined by GATK. All final candidate variants were manually reviewed by using the IGV browser as reported previously [13].

**Additional file 2:** **Table S1.** Targeted mutational analyses were performed in all three cases.

**Additional file 3:** **Table S2.** Details of primary antibodies used for immunohistochemical staining.

**Additional file 4:** **Figure S1.** More extensive morphologic and immunophenotypic features of Case 1. **Figure S2.** Immunoglobin gene (IG) rearrangement results from Case 1. **Figure S3.** More extensive morphologic and immunophenotypic features of Case 2. **Figure S4.** Immunoglobin gene (IG) rearrangement results from Case 2. **Figure S5. **Morphologic and immunophenotypic findings in bone marrow (BM) in June 2017 (Case 3). **Figure S6.** Morphologic and immunophenotypic findings of the submandibular gland core biopsy in May 2018 (Case 3). **Figure S7.** Morphologic and immunophenotypic findings for BM biopsy in September 2018 (Case 3). **Figure S8.** Immunoglobin gene (IG) rearrangement results from Case 3 (using last lymph node resection).

## Data Availability

All data generated or analyzed during this study are included in this published article (and its supplementary information files).
