# Supplementary material for: Recognizing puzzling PD1 + infiltrates in marginal zone lymphoma by integrating clonal and mutational findings: pitfalls in both nodal and transformed splenic cases
Source: Diagn Pathol. 2023 Dec 11;18:134. doi: 10.1186/s13000-023-01422-9 (PMC10712042; doi:10.1186/s13000-023-01422-9)
Supplement: Supplementary file 1 — Additional file 1. Supplementary Materials. Methods. Histology and Immunohistochemistry. Cases were collected from the Peking University Cancer Hospital and reviewed by an expert panel (YF.S., YM.L., and XH.L.), with a consensus diagnosis of MZL or tSMZL. The study was approved by the ethical committee of the institution and followed the 1964 Helsinki Declaration. Hematoxylin and eosin (H&E) and immunohistochemistry-stained slides from each case were evaluated. Immunohistochemistry was performed on FFPE sections on a Ventana Benchmark automated immunostainer using UltraView detection kits. The panel of antibodies can be seen in Table S2. The interpretation of the PD1 staining pattern included both the predominant location of the PD1-positive cells (follicular or extrafollicular) and was considered “normal” if most PD-1-positive cells were confined to intrafollicular areas and concentrated in the light zone. In situ hybridization to detect EBV-encoded RNA. EBV status was determined by in situ hybridization (ISH) to detect EBV-encoded RNA 1 and 2 (EBER1/2s) using peroxidase-labeled probes (ISH-7001UM, Beijing Zhongshan Golden Bridge Biotechnology). Tissue from a known EBV-positive nasopharyngeal carcinoma was used as a positive control. The EBV status was considered positive if at least one definitive cell expressed EBER. All H&E, IHC and ISH slides were independently and dual assessed. Immunoglobulin Gene and T-Cell Receptor Gene Rearrangement Studies. DNA was extracted from FFPE tissue sections using a QIAGEN QIAamp DNA FFPE Tissue Kit according to the manufacturer’s protocol (QIAGEN, Germantown, MD). Polymerase chain reaction (PCR) for immunoglobulin gene (IGH and IGK loci) and T-cell receptor (TCR locus) rearrangements was performed using commercially available BIOMED-2 multiplex PCR kits (Righton Gene, Shanghai). PCR products were separated by capillary electrophoresis and subjected to GeneScan analysis for confirmation of the monoclonal character of the IG or TC [file 13000_2023_1422_MOESM1_ESM.docx]

**Supplementary Materials and methods
*Histology and Immunohistochemistry***
Cases were collected from the Peking University Cancer hospital, reviewed by an expert panel (YF.S., YM.L., and XH.L.), with a consensus diagnosis of MZLor tSMZL. The study was approved by the ethical committee of the institution and followed the 1964 Helsinki Declaration. Hematoxylin and eosin (H&E) and immunohistochemistry-stained slides from each case were evaluated. Immunohistochemistry was performed on FFPE sections on a Ventana Benchmark automated immunostainer using UltraView detection kits. The panel of antibodies can be seen in Table S2.

The interpretation of PD1 staining pattern included both the predominant location of the PD1-positive cells (follicular or extrafollicular), and was considered as “normal” if most PD-1 positive cells were confined to intrafollicular areas and concentrated in the light zone.

***In situ hybridization to detect EBV-encoded RNA***

EBV status was determined by in situ hybridization (ISH) to detect EBV-encoded RNA 1 and 2 (EBER1/2s) using peroxidase-labelled probes (ISH-7001UM, Beijing Zhongshan Golden Bridge Biotechnology). Tissue from a known EBV-positive nasopharyngeal carcinoma was used as a positive control. The EBV status was considered positive if at least one definitive cell expressed EBER.

All the H&E, IHC and ISH slides were independently dual-assessed.

***Immunoglobulin Gene and T-Cell Receptor Gene Rearrangement Studies***
DNA was extracted from FFPE tissue sections using QIAGEN QIAamp DNA FFPE Tissue Kit according to the manufacturer’s protocol (QIAGEN, Germantown, MD). Polymerase chain reaction (PCR) for immunoglobulin gene (IGH and IGK loci) and T-cell receptor (TCR locus) rearrangements was performed using commercially available BIOMED-2 multiplex PCR kits (Righton Gene, ShangHai). PCR products were separated by capillary electrophoresis, and were subjected to GeneScan analysis for confirmation of the monoclonal character of the IG or TCR gene rearrangements on an ABI 9700Genetic Analyzer (Applied Biosystems, Foster City, CA), and electropherograms were analyzed using the GeneMapper software, version 4.0. ***Targeted-Exome-Sequencing (TES) and sequence data analysis***

The genomic DNA (gDNA) extraction from FFPE tissues, Library preparation, target gene enrichment was done according to the manufacturer’s protocol. The gDNA libraries were subjected to high-throughput sequencing with 150-bp pair-end reads on the NovaSeq 60,000 Sequencing System (Illumina, San Diego, CA) supported by a commercial vendor (Geneplus-Beijing, China). The average sequencing depth of tissues was ～500×. Sequence reads were aligned using BWA version 0.5.9 (Broad Institute). Single nucleotide variants (SNVs) were called using MuTect (version 1.1.4) and NChot. Small insertions and deletions (Indels) were determined by GATK. All final candidate variants were manually reviewed by using the IGV browser, as reported previously [1].

**Reference**

1. Ye Y, Ding N, Mi L, Shi Y, Liu W, Song Y *et al*: **Correlation of mutational landscape and survival outcome of peripheral T-cell lymphomas**. *Exp Hematol Oncol* 2021, **10**(1):9.
