# Supplementary material for: Recognizing puzzling PD1 + infiltrates in marginal zone lymphoma by integrating clonal and mutational findings: pitfalls in both nodal and transformed splenic cases
Source: Diagn Pathol. 2023 Dec 11;18:134. doi: 10.1186/s13000-023-01422-9 (PMC10712042; doi:10.1186/s13000-023-01422-9)
Supplement: Supplementary file 2 — Additional file 2: Table S1. Targeted mutational analyses were performed in all three cases. [file 13000_2023_1422_MOESM2_ESM.docx]

| **Case** | **Gene Symbol** | **Exon** | **AAChange** | **列1** | **AF** |
| --- | --- | --- | --- | --- | --- |
| Case 1 | KMT2D | exon39 | c.11878C>T(p.Q3960*) | stop_gained | 25.9% |
| Case 1 | KMT2D | exon39-41 | c.13528_13839+6del | Large_fragment_deletion | 17.5% |
| Case 1 | TNFAIP3 | exon6 | c.930dup(p.E311Rfs*22) | Truncating | 3.8% |
| Case 1 | NOTCH2 | exon34 | c.6907_6914del(p.P2303Dfs*7) | Truncating | 22.9% |
| Case 1 | TBL1XR1 | exon8 | c.756G>A(p.W252*) | stop_gained | 22.8% |
| Case 2 | EP300 | exon27 | c.4396T>C (p.W1466R) | missense | 8.8% |
| Case 2 | KMT2C | exon7 | c.962G>A (p.S321N ) | missense | 9.1% |
| Case 2 | KMT2D | exon48 | c.15575delT (p.L5192fs) | Truncating | 10.6% |
| Case 2 | KMT2D | exon39 | c.11360_11361insGTCCCCTCAA(p.Q3788fs ) | Truncating | 3.1% |
| Case 2 | KMT2D | exon34 | c.9651_9652delTG (p.S3217fs) | Truncating | 4.2% |
| Case 3 | TNFAIP3 | exon3 | c.400_401del(p.D134fs) | Truncating | 5.5% |
| Case 3 | CD58 | exon3 | c.C454T(p.R152X) | missense | 7.1% |
| Case 3 | KMT2D | exon32 | c.C8047T(p.R2683C) | missense | 5.9% |

**Table S1 Targeted mutational analyses were performed in all three cases**
