# Supplementary material for: Recognizing puzzling PD1 + infiltrates in marginal zone lymphoma by integrating clonal and mutational findings: pitfalls in both nodal and transformed splenic cases
Source: Diagn Pathol. 2023 Dec 11;18:134. doi: 10.1186/s13000-023-01422-9 (PMC10712042; doi:10.1186/s13000-023-01422-9)
Supplement: Supplementary file 3 — Additional file 3: Table S2. Details of primary antibodies used for immunohistochemical staining. [file 13000_2023_1422_MOESM3_ESM.docx]

Table S2 Details in primary Antibodies in immunohistochemical staining.

| **Primary antibody** | **clone** | **Manufacturer** | **Specified Target** | **location** | **Dilution** |
| --- | --- | --- | --- | --- | --- |
| BCL6 | ZR280 | Beijing Xiya Golden Bridge Biotechnology | TFH cell or Tumor cell | cell nuclear | 1∶50 |
| CD3_ε_ | A0452 | Dako, Agilent technologies, Santa Clara, USA | T cell or Tumor cell | cytoplasm | 1∶50 |
| CD10 | 56C6 | Dako, Agilent technologies, Santa Clara, USA | TFH cell or Tumor cell | cell membrane | 1∶50 |
| CD20 | L26 | Dako, Agilent technologies, Santa Clara, USA | B cell | cell membrane | 1∶50 |
| PAX 5 | ZP007 | Thermo Fisher, Rockford, USA | B cell | cell nuclear | 1∶50 |
| BCL2 | 124 | Dako, Agilent technologies, Santa Clara, USA | TFH cell or Tumor cell | cytoplasm | 1∶50 |
| CD21 | EP64 | Beijing Zhongshan Golden Bridge Biotechnology | FDC cell | cell nuclear | 1∶50 |
| CD30 | JCM182 | Novocastra, Leica Biosystems, UK | Immunoblastic Cell | cell nuclear | 1∶50 |
| CD4 | UMAB64 | Beijing Xiya Golden Bridge Biotechnology | T helper cell or Tumor cell | cell membrane | 1∶50 |
| CD8 | 4B11 | Novocastra, Leica Biosystems, UK | Cytotoxic T-cell or Tumor cell | cell membrane | 1∶50 |
| CXCL13 | 53610 | R&D Systems | T cell or Tumor cell | cytoplasm | 1∶50 |
| Ki67 | MIB1 | Dako, Agilent technologies, Santa Clara, USA | Tumor cell | cell nuclear | 1∶50 |
| PD1 | UMAB199 | Beijing Zhongshan Golden Bridge Biotechnology | TFH cell or Tumor cell | cell nuclear | 1∶50 |

Note：Follicular T helper cells（TFH），Follicular Dendritic Cell（FDC）
