# Supplementary material for: Recognizing puzzling PD1 + infiltrates in marginal zone lymphoma by integrating clonal and mutational findings: pitfalls in both nodal and transformed splenic cases
Source: Diagn Pathol. 2023 Dec 11;18:134. doi: 10.1186/s13000-023-01422-9 (PMC10712042; doi:10.1186/s13000-023-01422-9)
Supplement: Supplementary file 4 — Additional file 4: Figure S1. More extensive morphologic and immunophenotypic features of Case 1. Figure S2. Immunoglobin gene (IG) rearrangement results from Case 1. Figure S3. More extensive morphologic and immunophenotypic features of Case 2. Figure S4. Immunoglobin gene (IG) rearrangement results from Case 2. Figure S5. Morphologic and immunophenotypic findings in bone marrow (BM) in June 2017 (Case 3). Figure S6. Morphologic and immunophenotypic findings of the submandibular gland core biopsy in May 2018 (Case 3). Figure S7. Morphologic and immunophenotypic findings for BM biopsy in September 2018 (Case 3). Figure S8. Immunoglobin gene (IG) rearrangement results from Case 3 (using last lymph node resection). [file 13000_2023_1422_MOESM4_ESM.docx]

**Figure S1 More extensive morphologic and immunophenotypic features of case 1.**

In lower power field, the nodular architecture was mostly effaced by neoplastic lymphoma cells in (A); by immunostaining, the tumor cells were positive for CD20(B) and PAX5(C) with vague nodular patten, and the CD21 positive follicular dendritic cells meshwork was retained(D). Peripherally infiltrated T cells manifested by immunostaining of CD3(E) and were positive were PD1(F). In high power field, the neoplastic B cell nodules, most cells were small to medium in size (G) were positive for CD20(H) , and CD21 positive follicular dendritic meshwork were partially effaced in this nodule(I), and peripherally infiltrating T cells were positive for CD3(J), and CD4 positive T cells(K) were more than CD8 positive cells(L), and were positive were PD1(M), but negative for CD10(N) and BCL6(O).

**
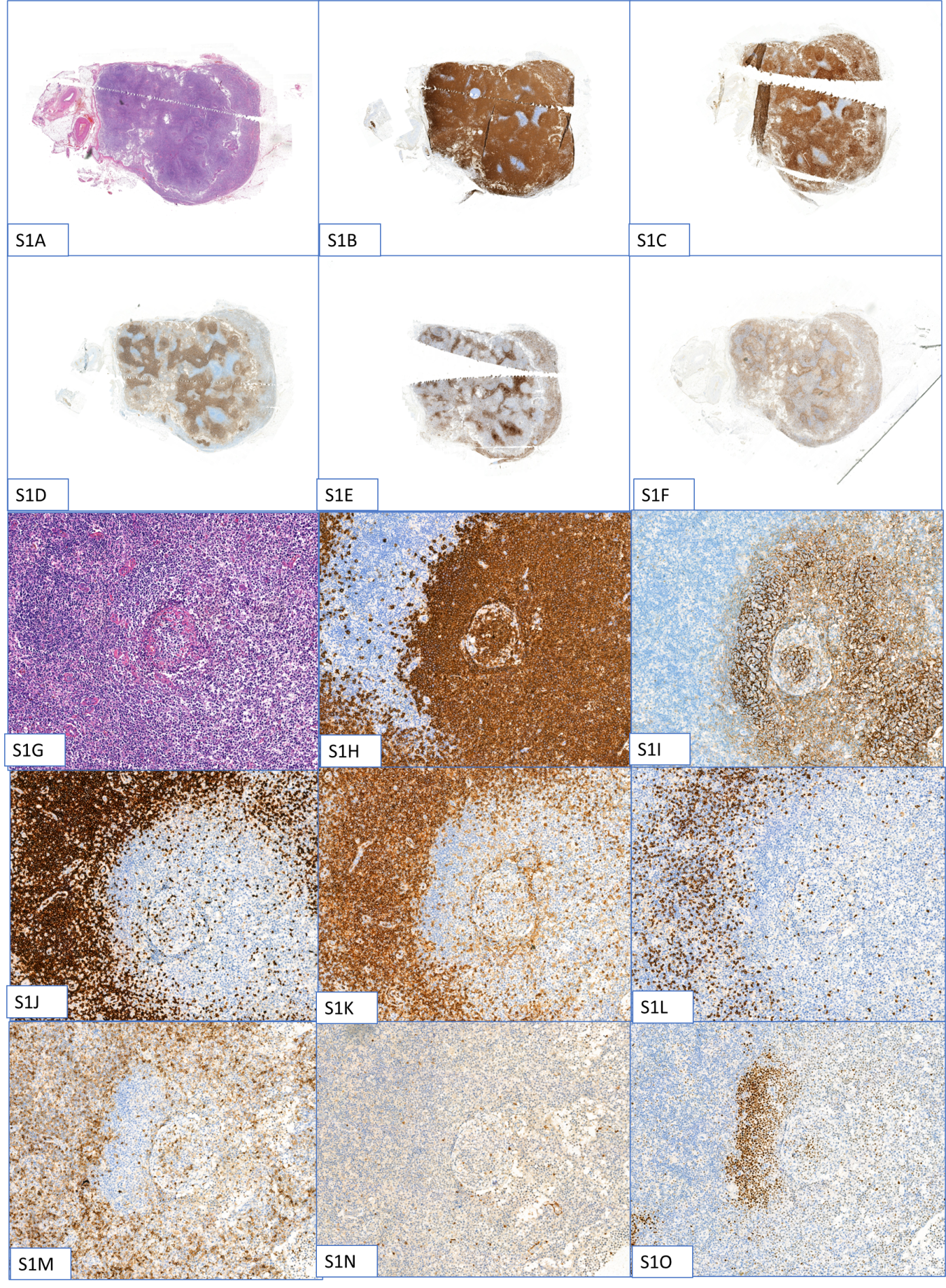
**

**Figure S2 Immunoglobin Gene (IG) rearrangement results for case 1**

Monoclonal results were seen in IGH -C (FR3-JH) tube, IGH -E (DH7-JH) tube, IGK -A (Vk-Jk) tube and IGK -B (Vk-Kde+intron-Kde) tube by Genescan method (1A -1D. BIOMED-2).


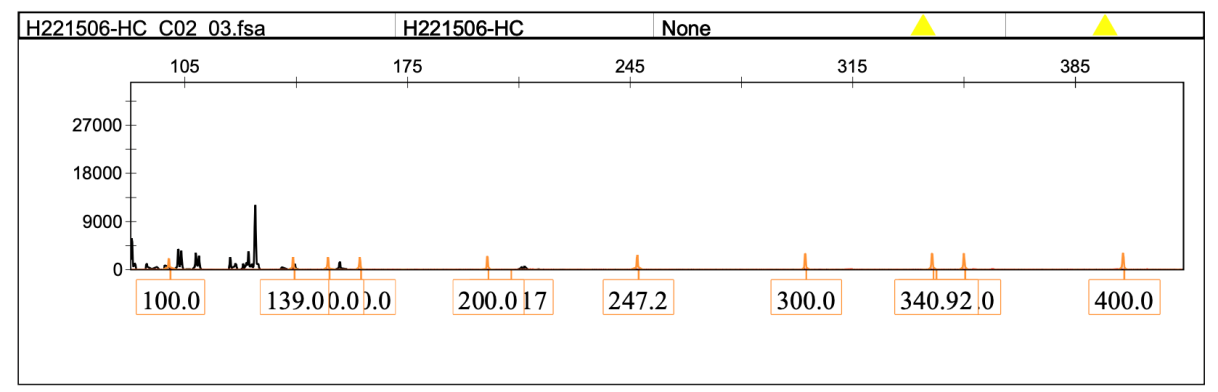


S2A


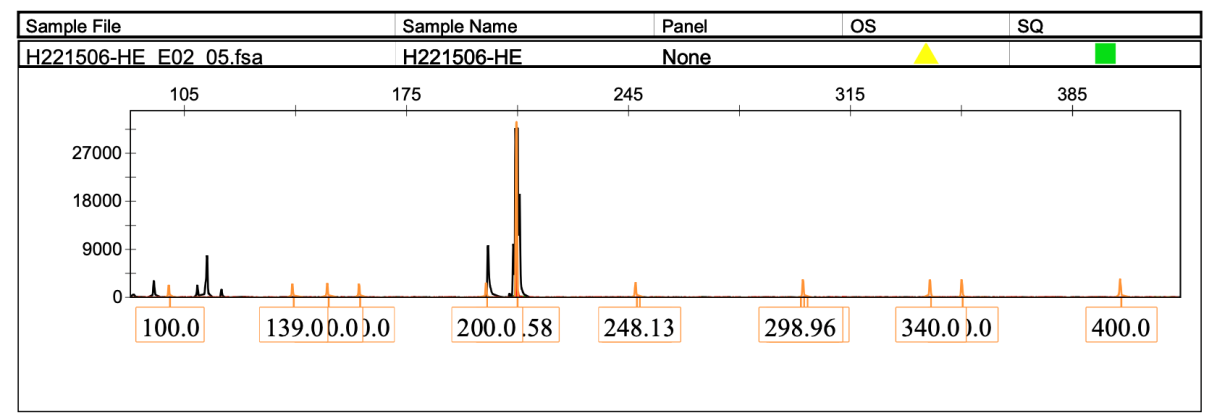


S2B


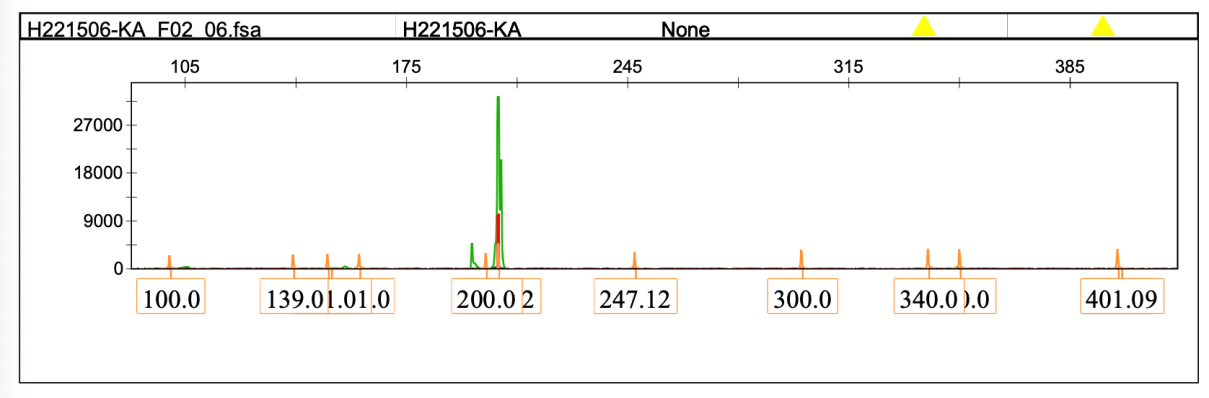


S2C


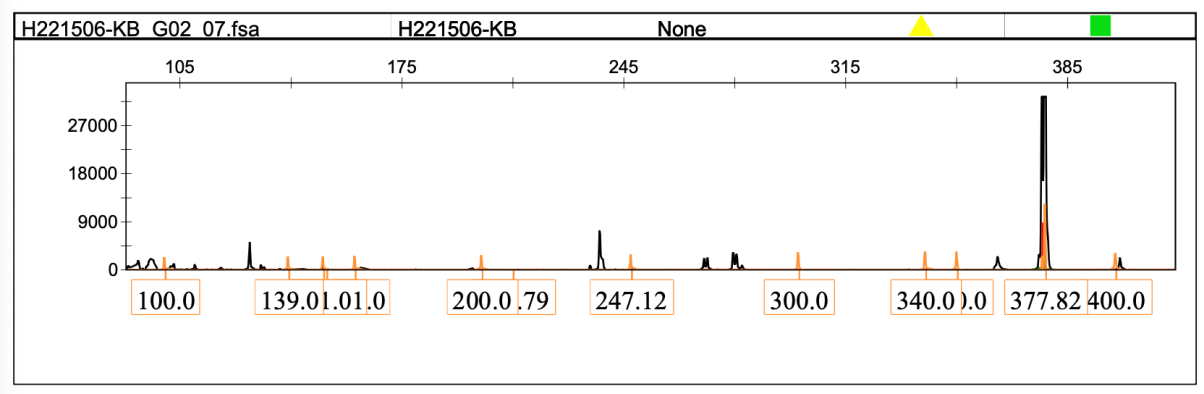


S2D

**Figure S3 More extensive morphologic and immunophenotypic features of case 2.**

In lower power field, the nodular architecture was also mostly effaced by neoplastic lymphoma cells in (A); by immunostaining, the tumor cells were positive for CD20(B) and PAX5(C) with vague nodular patten, and the CD21 positive follicular dendritic cells meshwork was strictly retained(D). Peripherally infiltrated T cells manifested by immunostaining of CD3(E) and were positive for PD1(F). In high power field, the neoplastic B cell nodules, most cells were small to medium in size (G) were positive for CD20(H) , and CD21 positive follicular dendritic meshwork were irregularly effaced in this nodule(I), and peripherally infiltrating T cells were positive for CD3(J), and most T cells were positive for CD4 (K), PD1(L), CXCL13(M)and partially positive for BCL6(N). but negative for CD10(O).


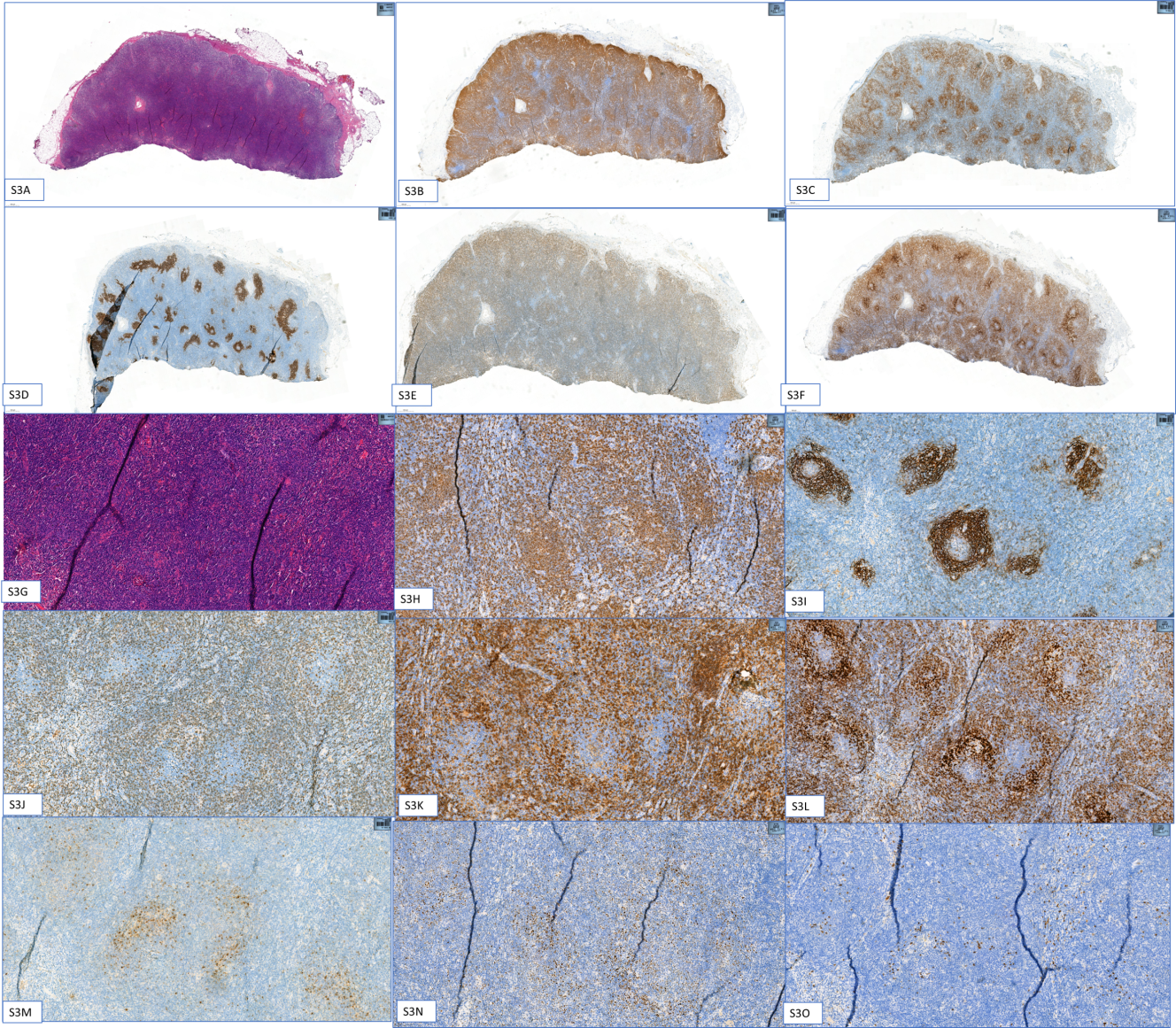


**Figure S4 Immunoglobin Gene (IG) rearrangement results for case 2** Monoclonal results were seen in IGH -A (FR1-JH) tube, IGH -C (FR3-JH) tube, IGH -E (DH7-JH) tube, IGK -A (Vk-Jk) tube and IGK -B (Vk-Kde+intron-Kde) tube ,IGL (Vλ-Jλ) tube by Genescan method (**2A-2F**,BIOMED-2 Method).


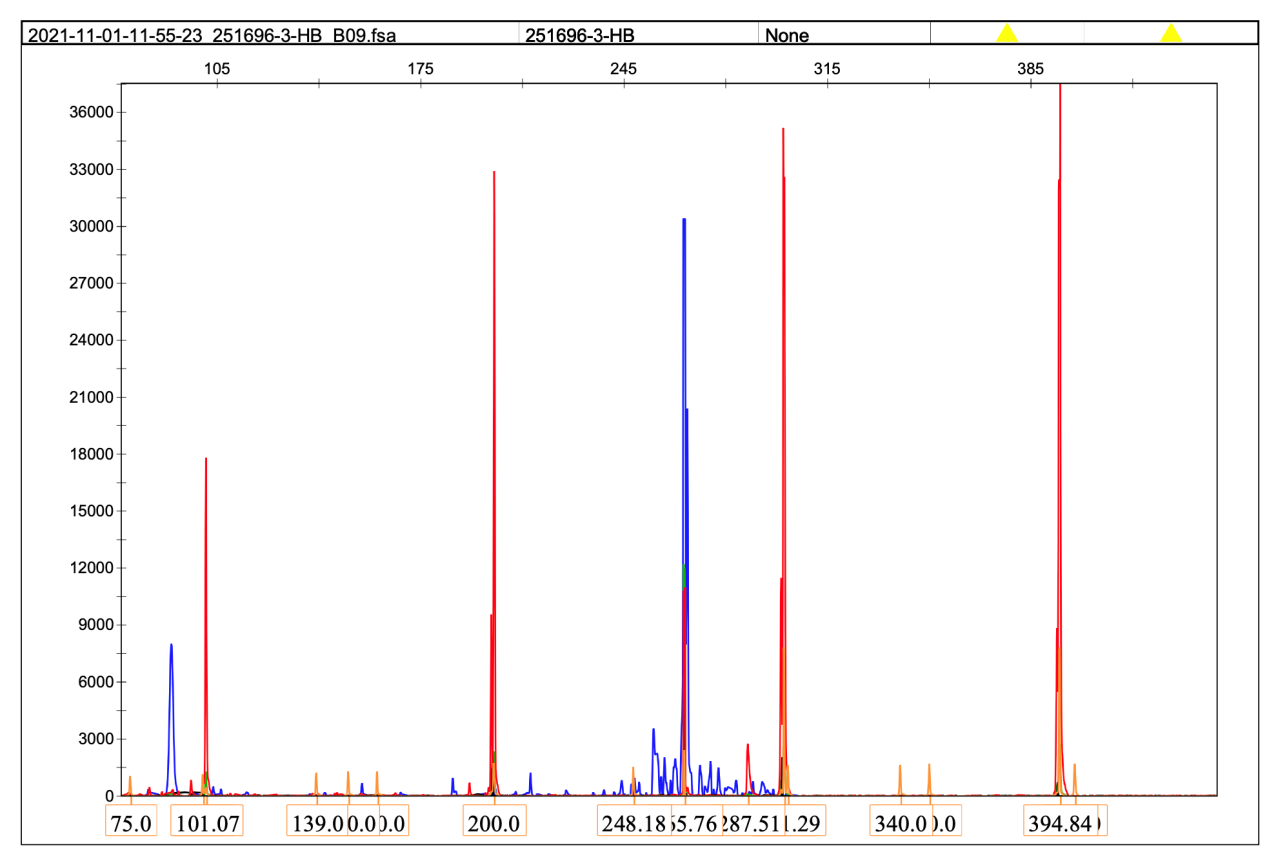


S4A


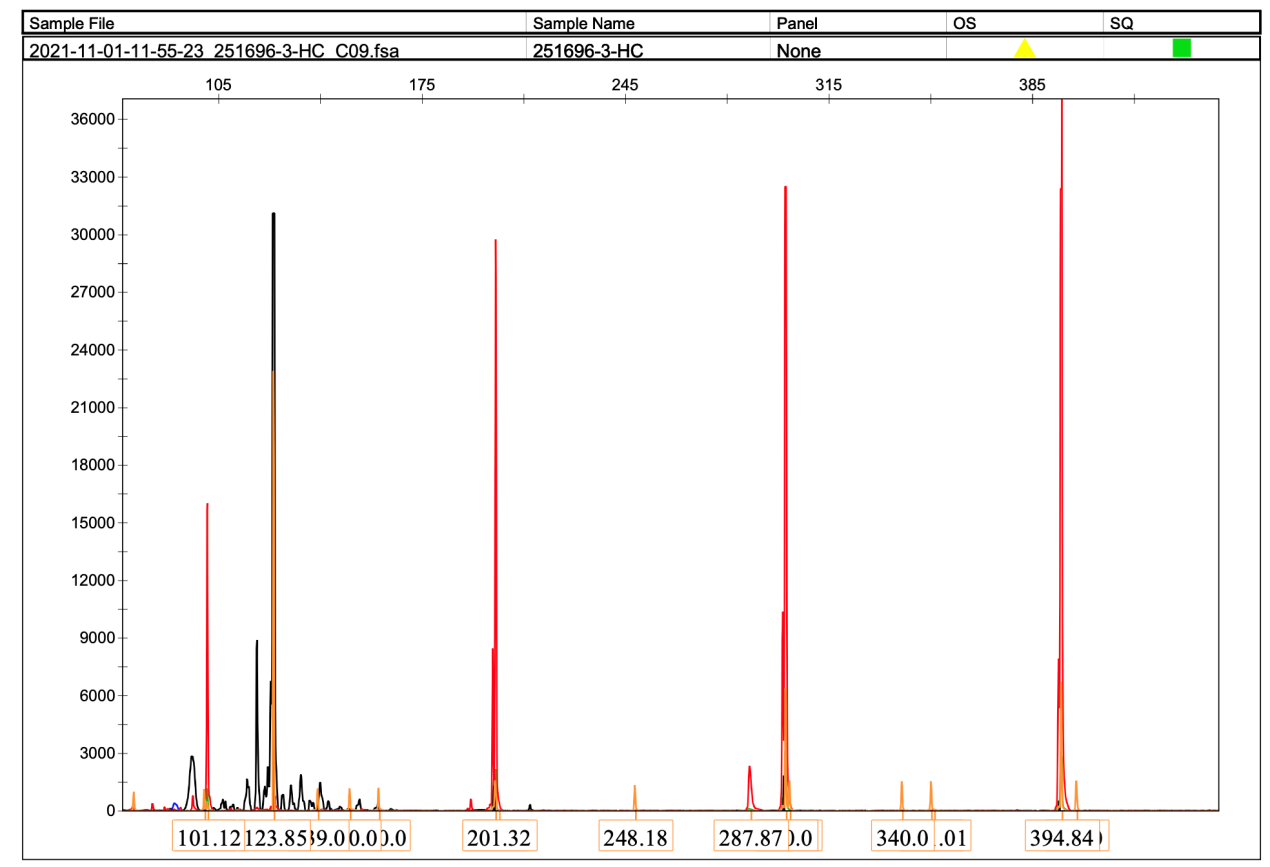


S4B


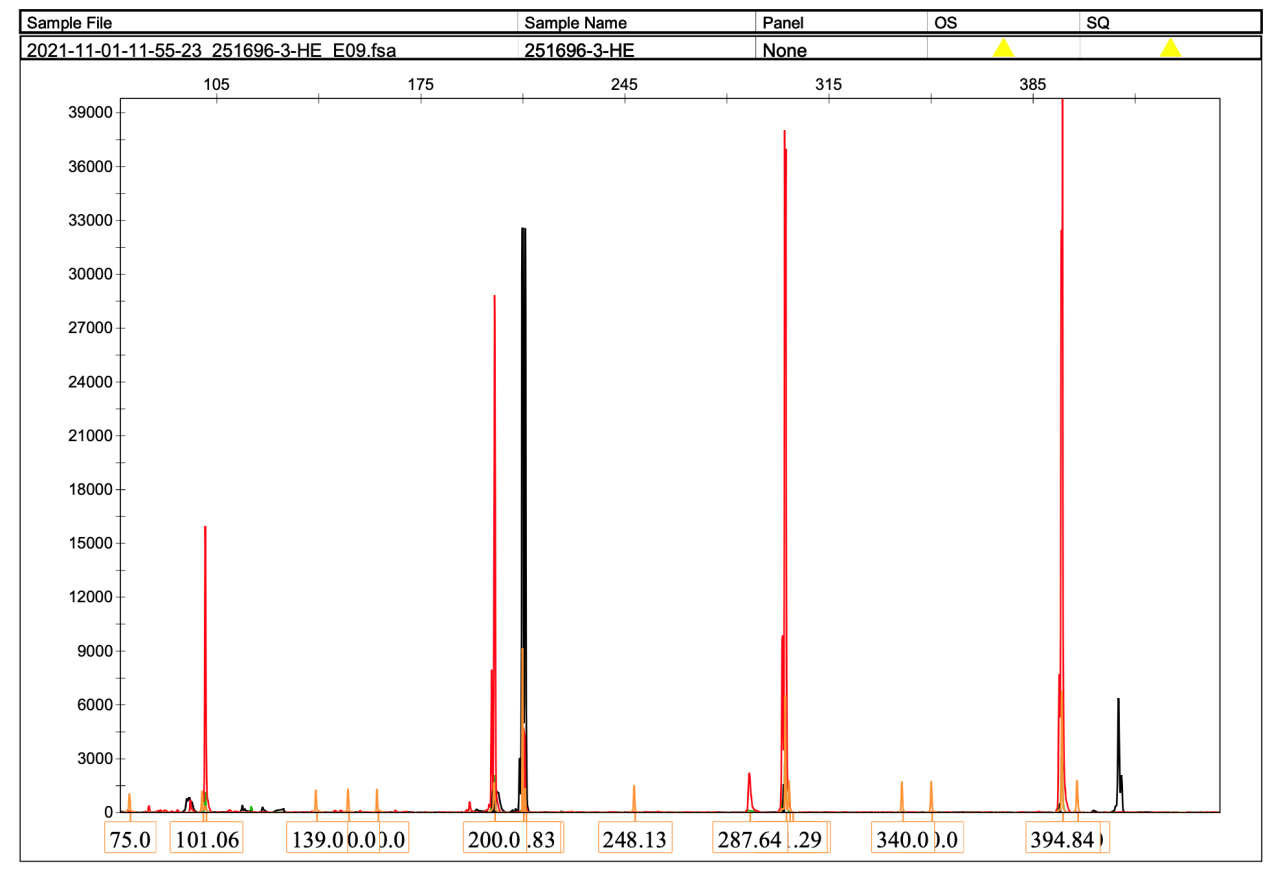


S4C


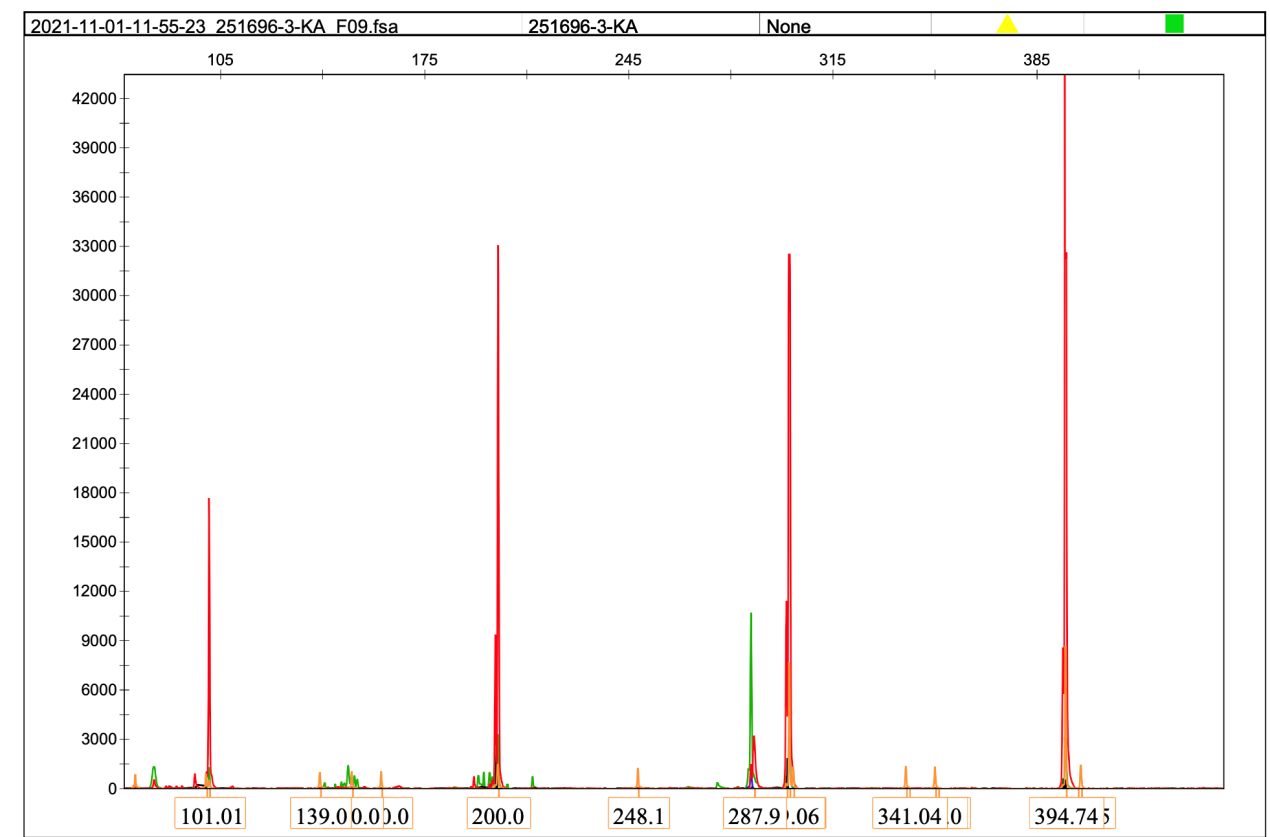


S4D


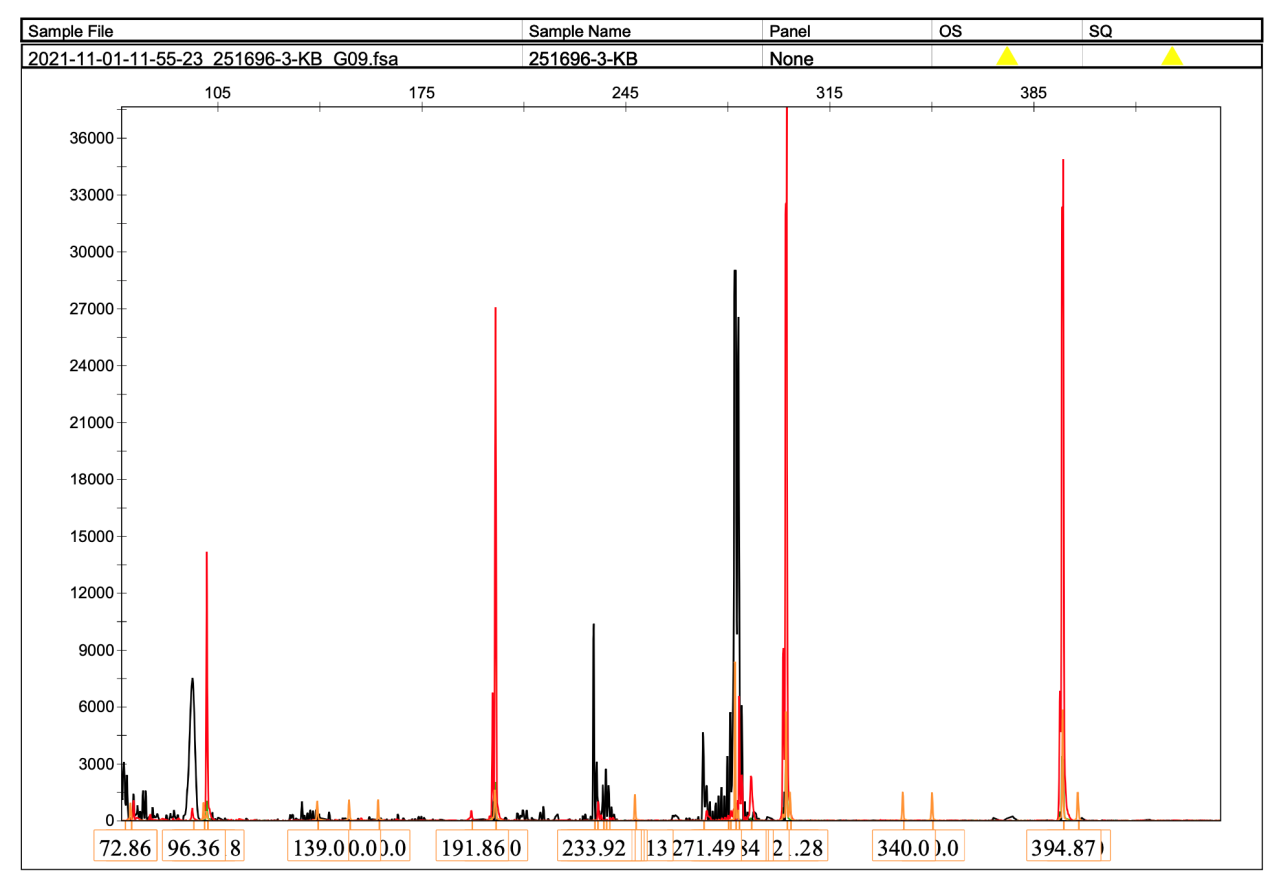


S4E


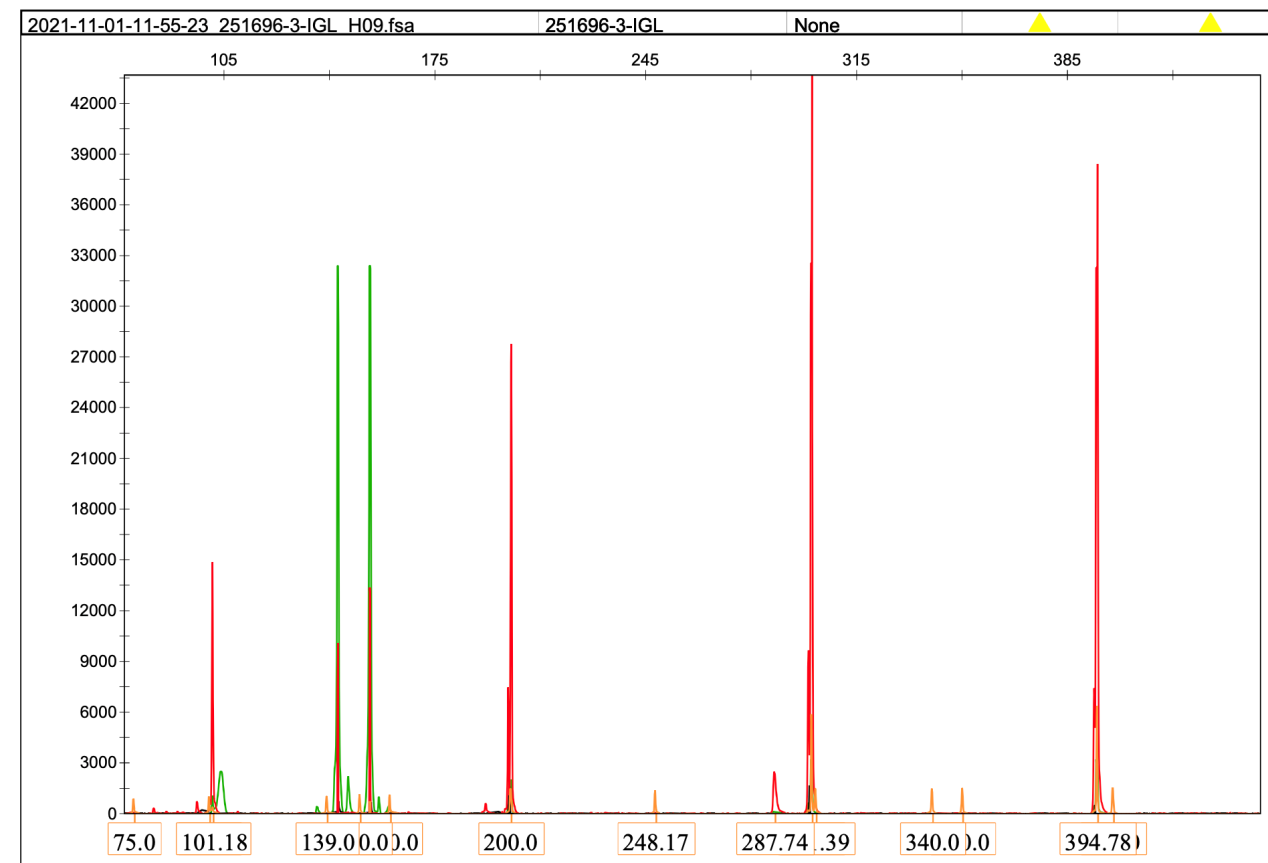


S4F

**Figure S5 Morphologic and immunophenotypic findings in bone marrow (BM) in June 2017 for Case 3.** Similar lymphocyte nodules can be seen in (**A)** and (**B**), with CD3+ T cell located centrally in(**C**) and CD20+ cells located peripherally in (**D**) of the neoplastic nodules.

**
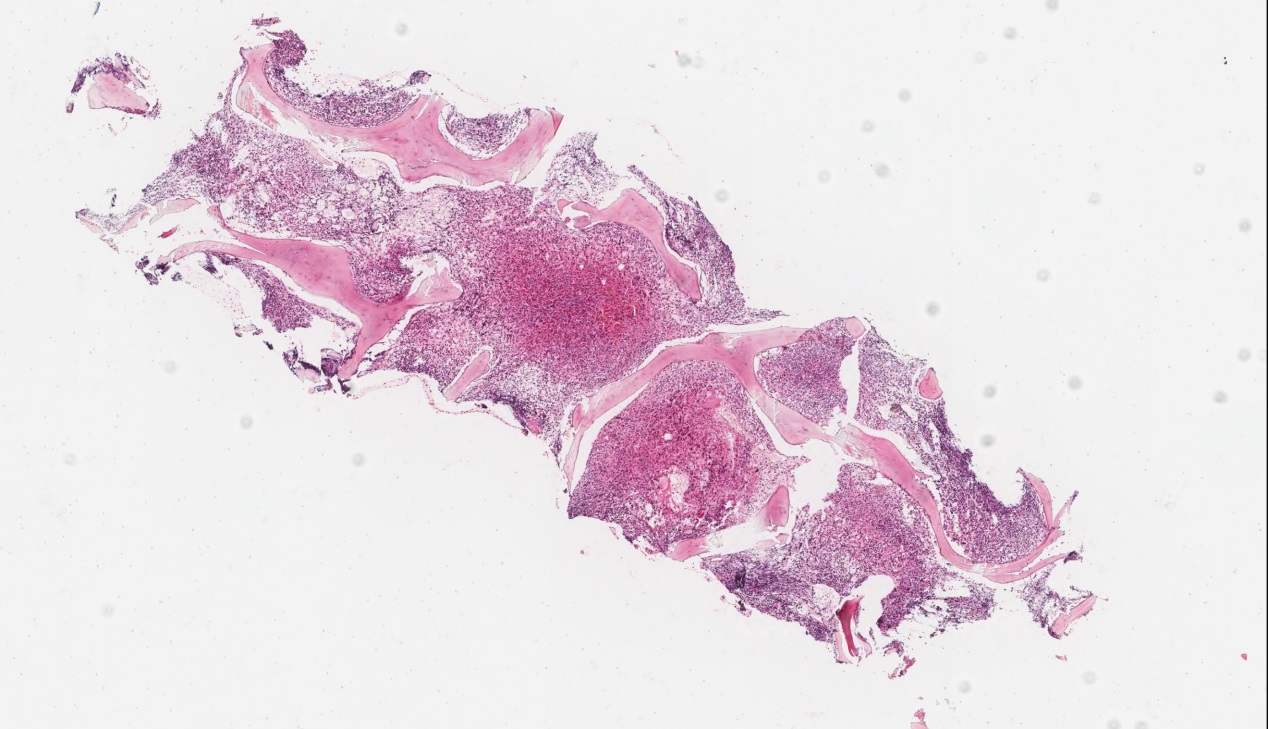
**

S5A

**
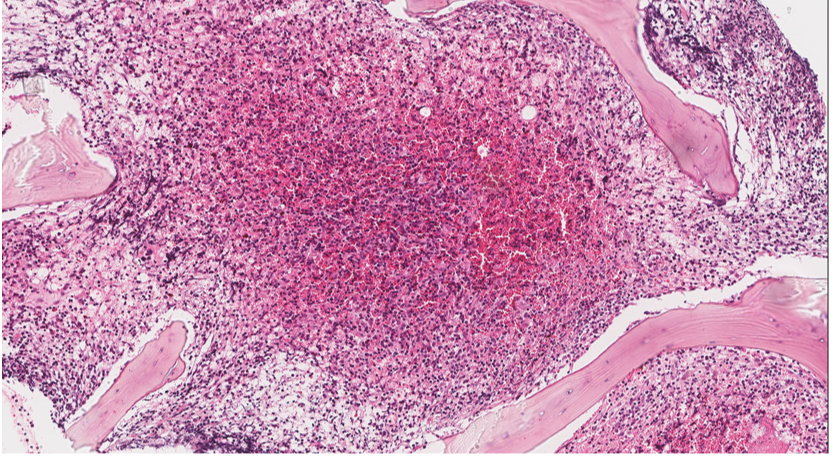
**

S5B

**
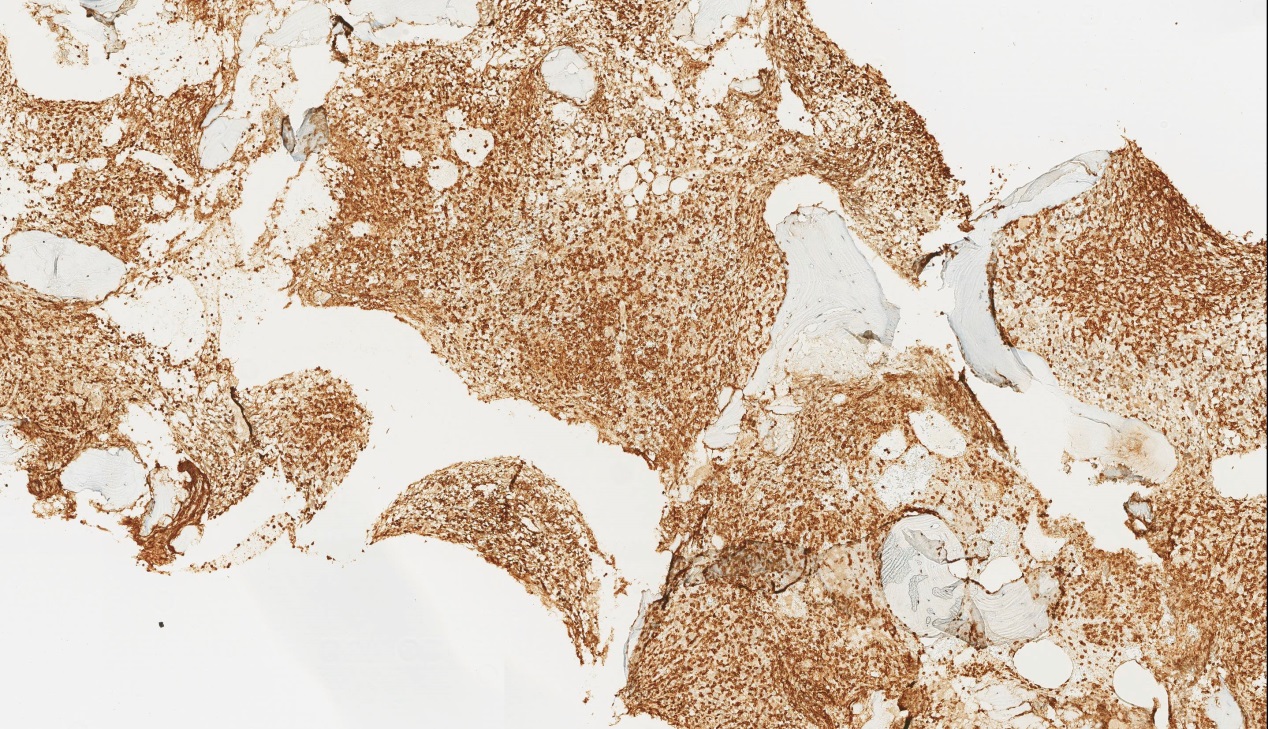
**

S5C

**
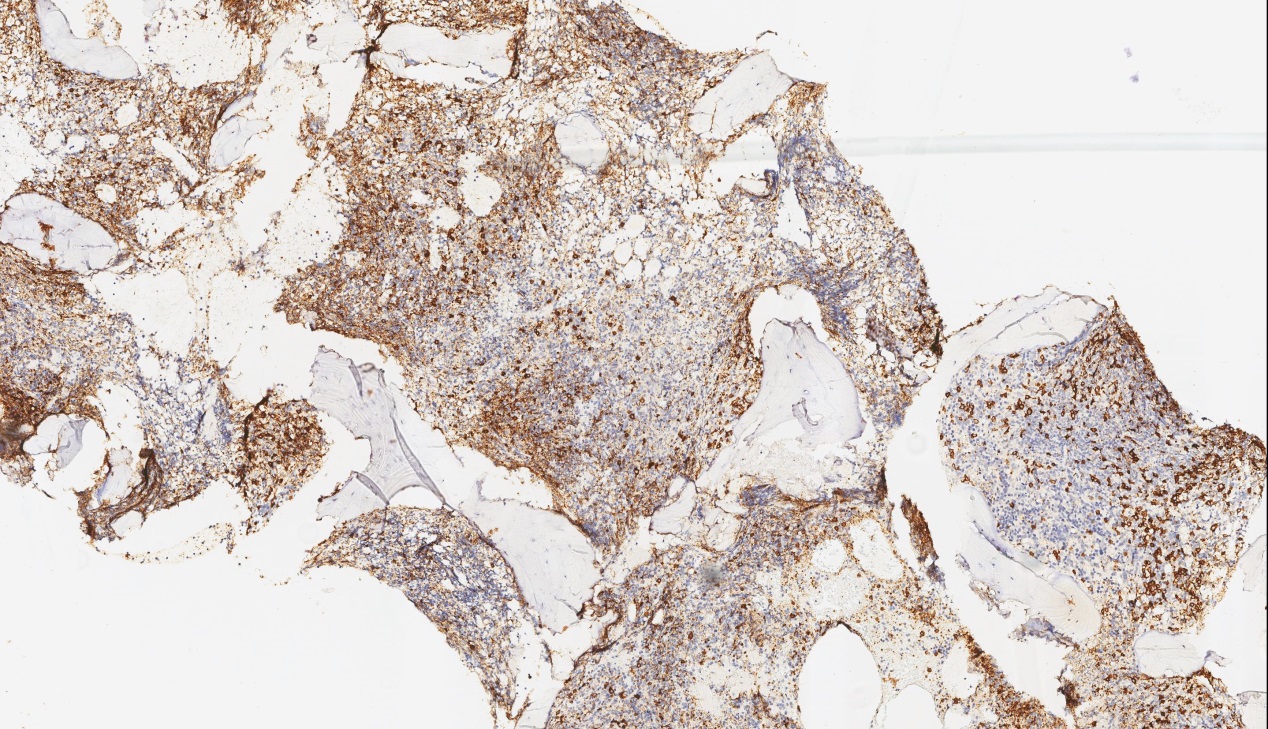
**

S5D

**Figure S6 Morphologic and immunophenotypic findings the submandibular gland core biopsy in May 2018 (Case 3).** Extensive accumulation and infiltration of atypical lymphocytes without nodular pattern were noted, most cells were small to medium with rare immunoblastic-like cells（**A** and **B**）, most were CD3+ smaller T cells (**C**), and there were some scattered larger CD20 positive cells (**D**), with Ki67 about 50% (**E**).

**
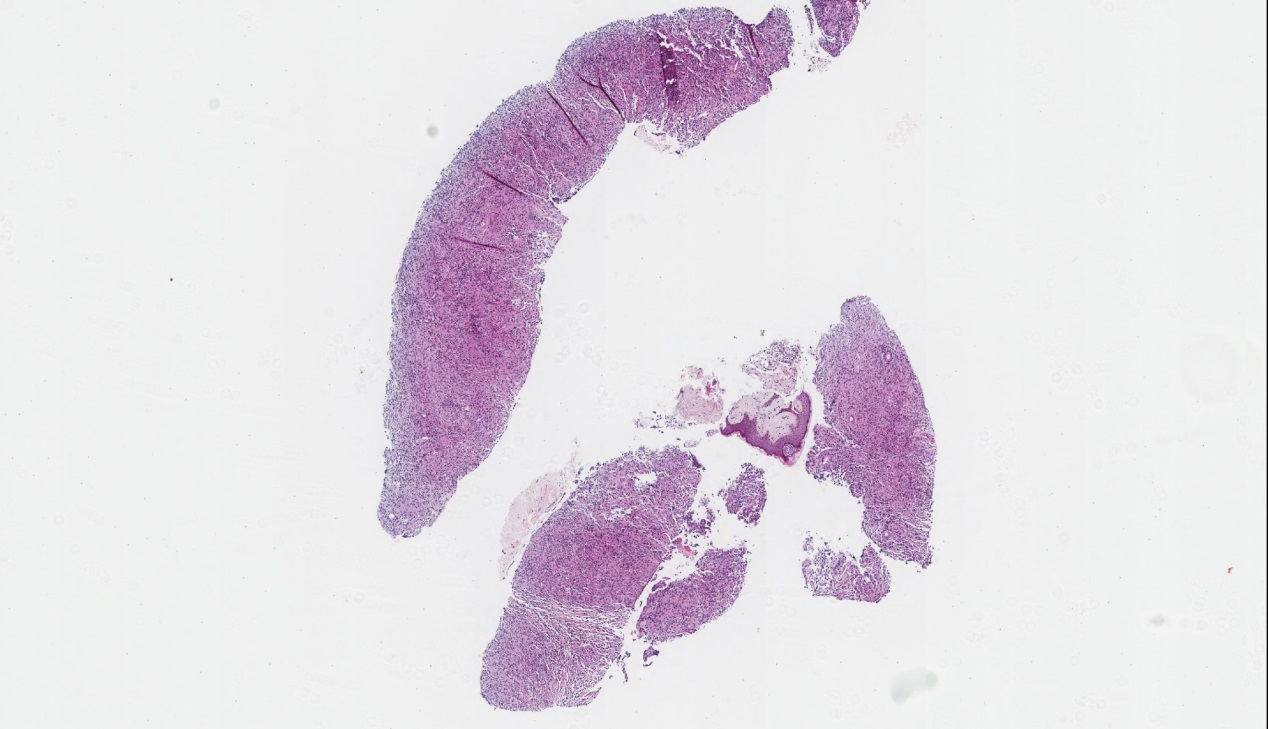
**

S6A

**
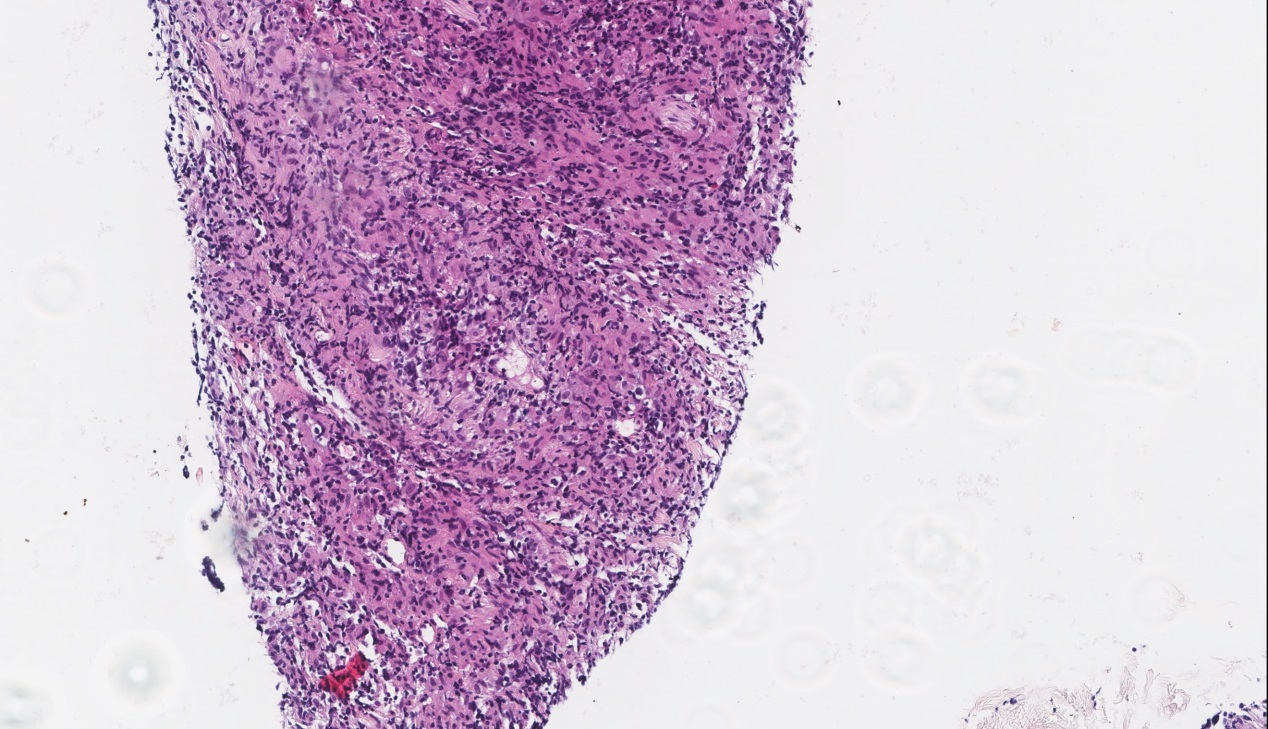
**

S6B

**
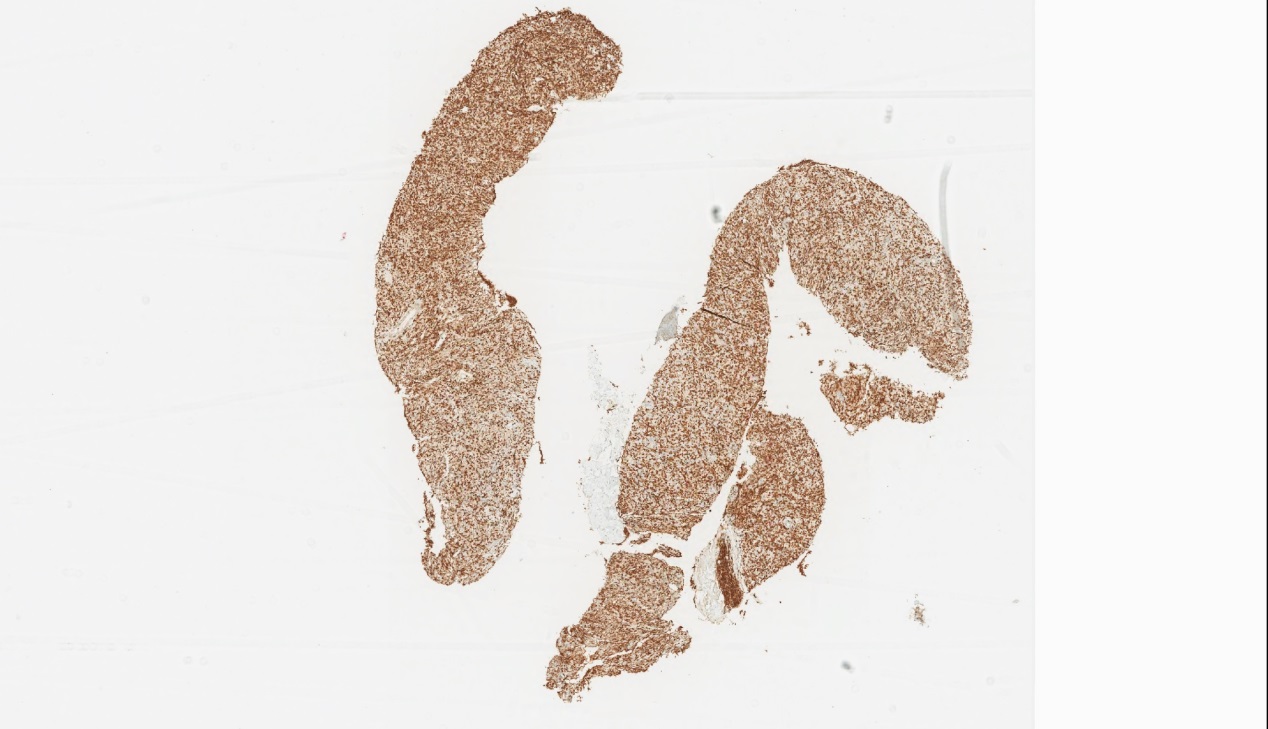
**

S6C

**
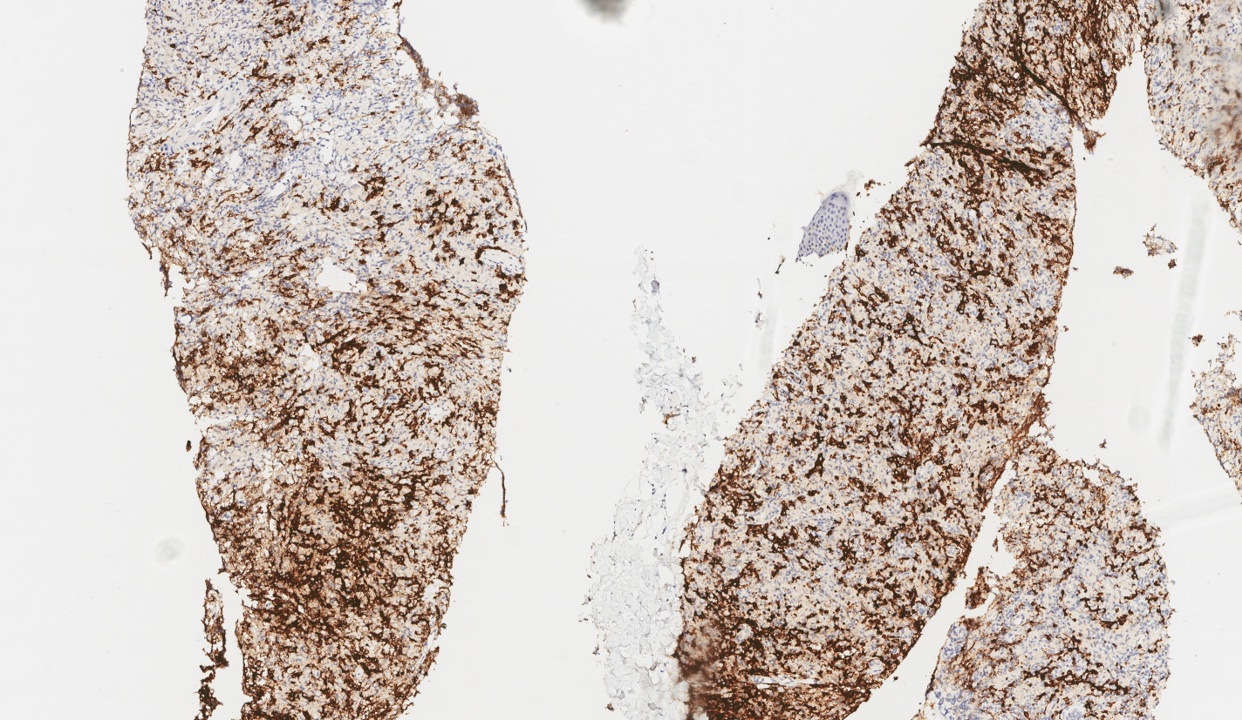
**

S6D

**
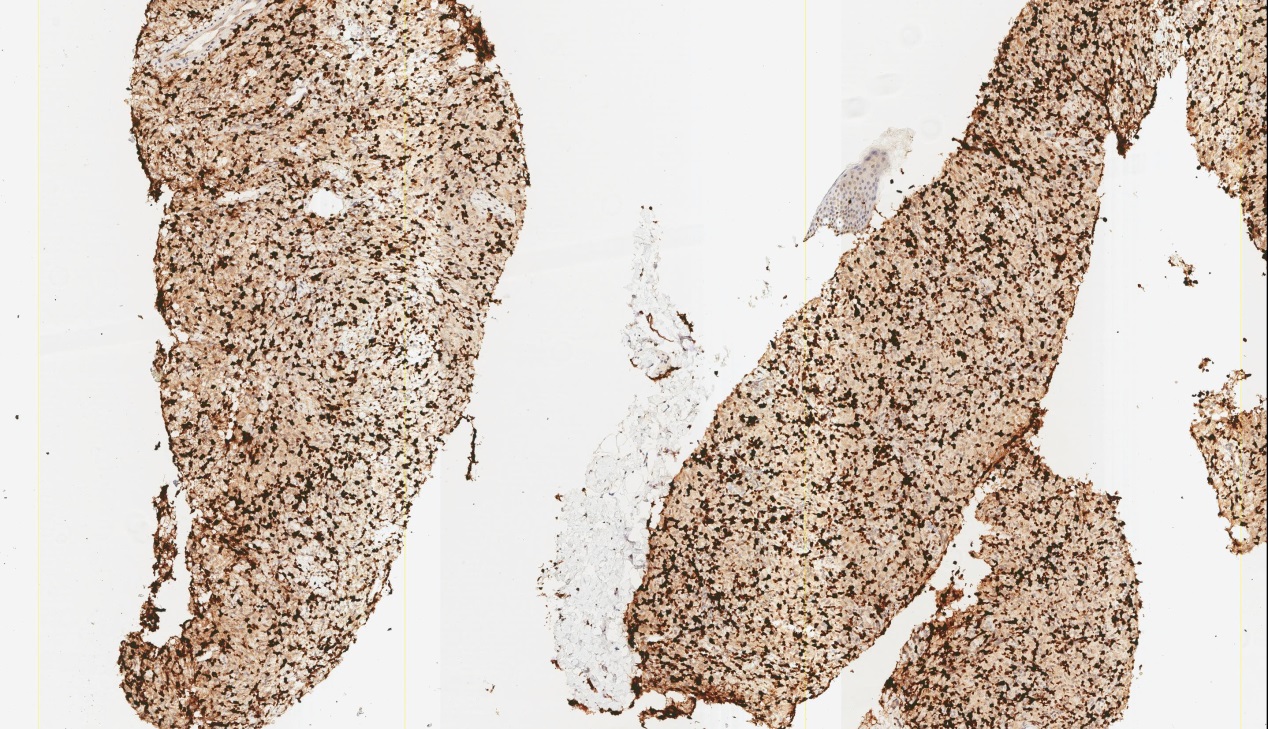
**

S6E

**Figure S7 Morphologic and immunophenotypic findings for BM biopsy in September 2018 (Case 3).** There were fewer atypical lymphocyte nodules from (**A**), cells in the center were CD3 positive (**B**)

**
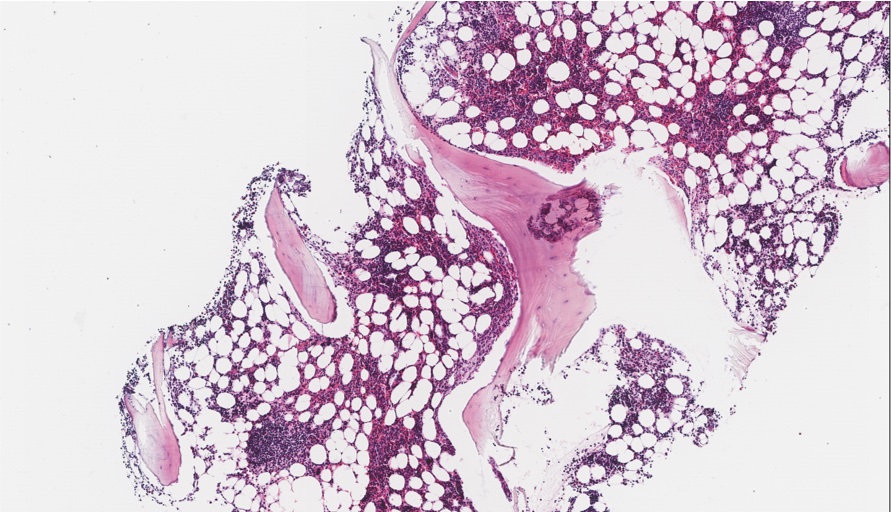
**

S7A

**
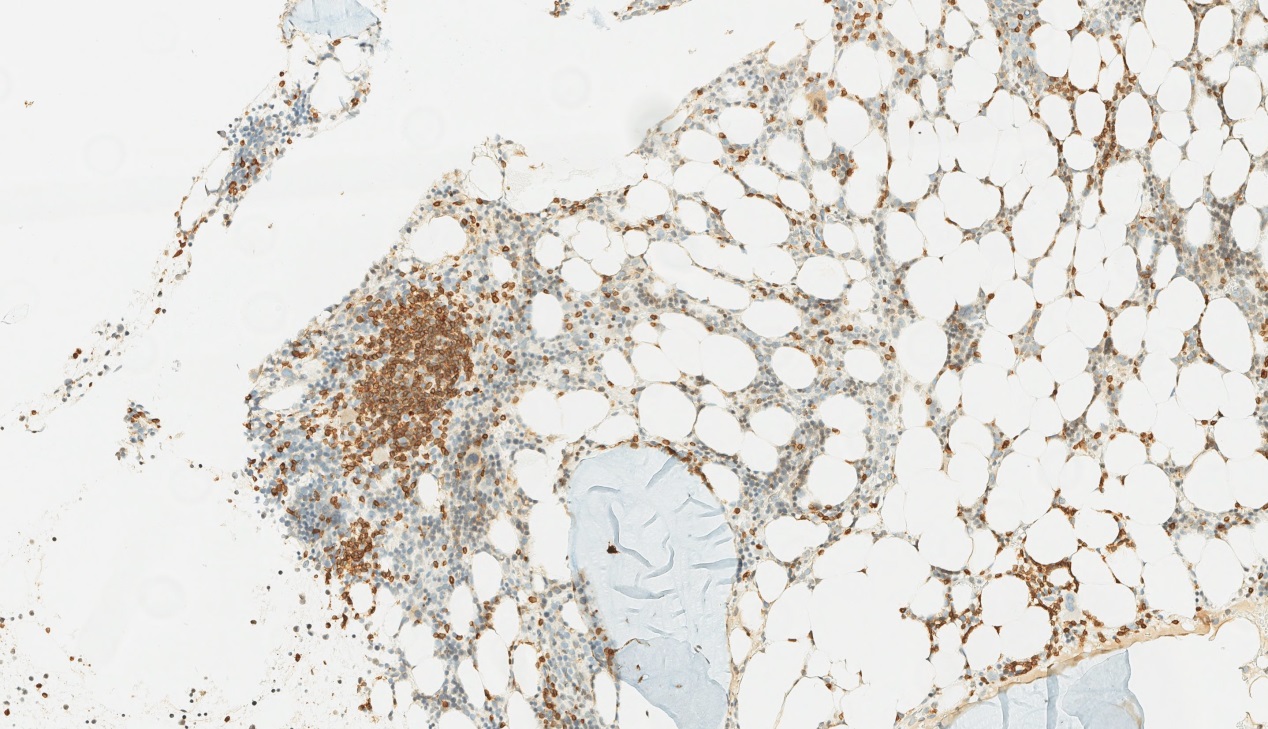
**

S7B

**Figure S8 Immunoglobin Gene (IG) rearrangement results for case 3 (using last lymph node resection)** Monoclonal results were seen in IGH -C (FR3-JH) tube, IGK -A (Vk-Jk) tube and IGK -B (Vk-Kde+intron-Kde) tube (**A-C**) by Genescan method (BIOMED-2).


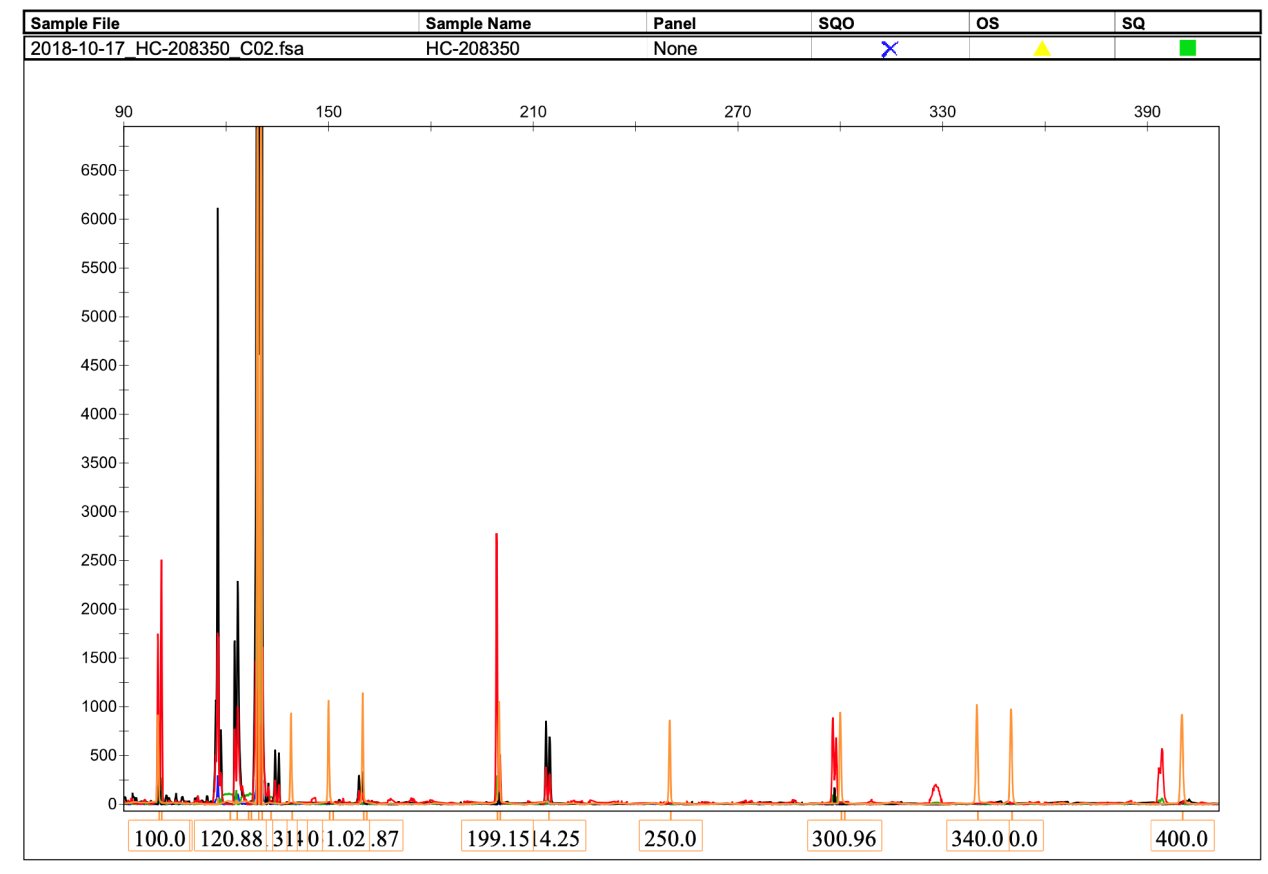


S8A


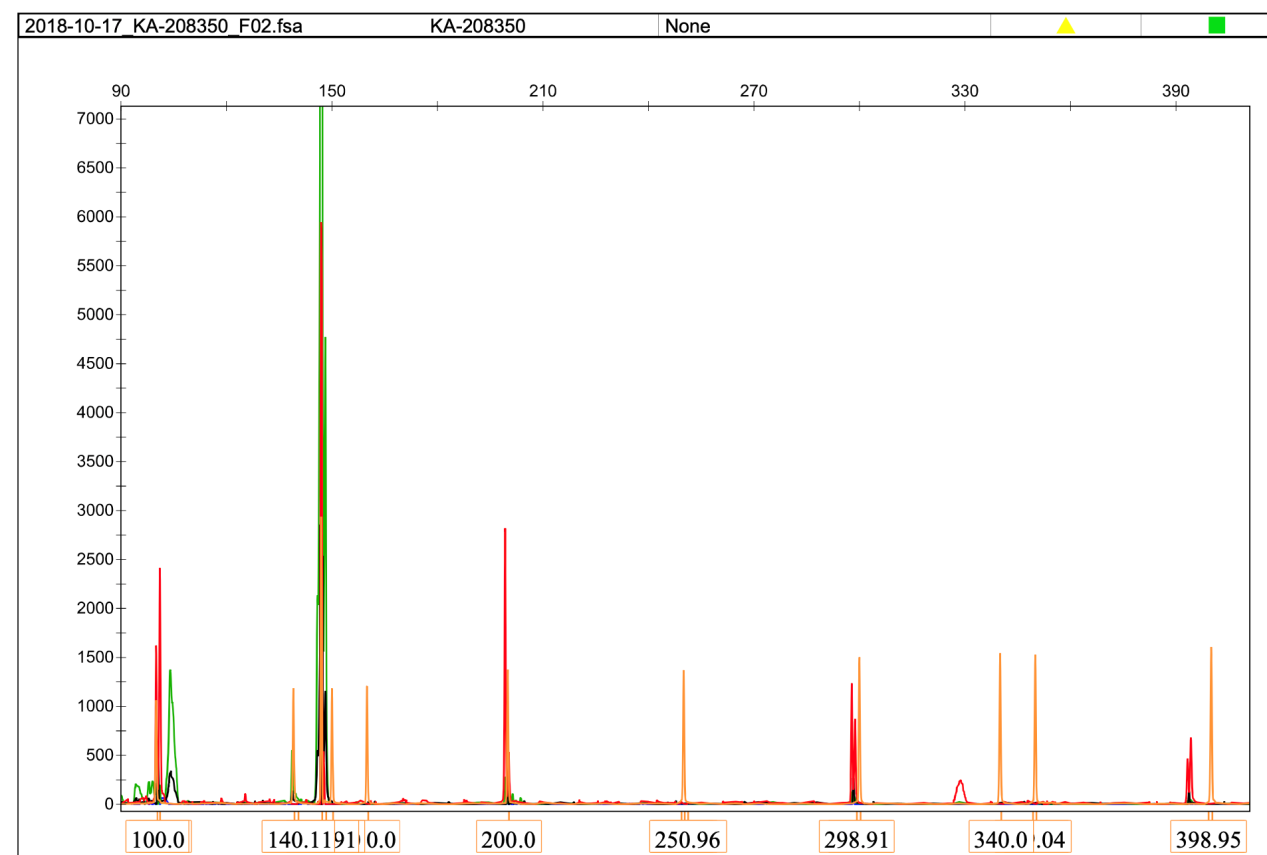


S8B


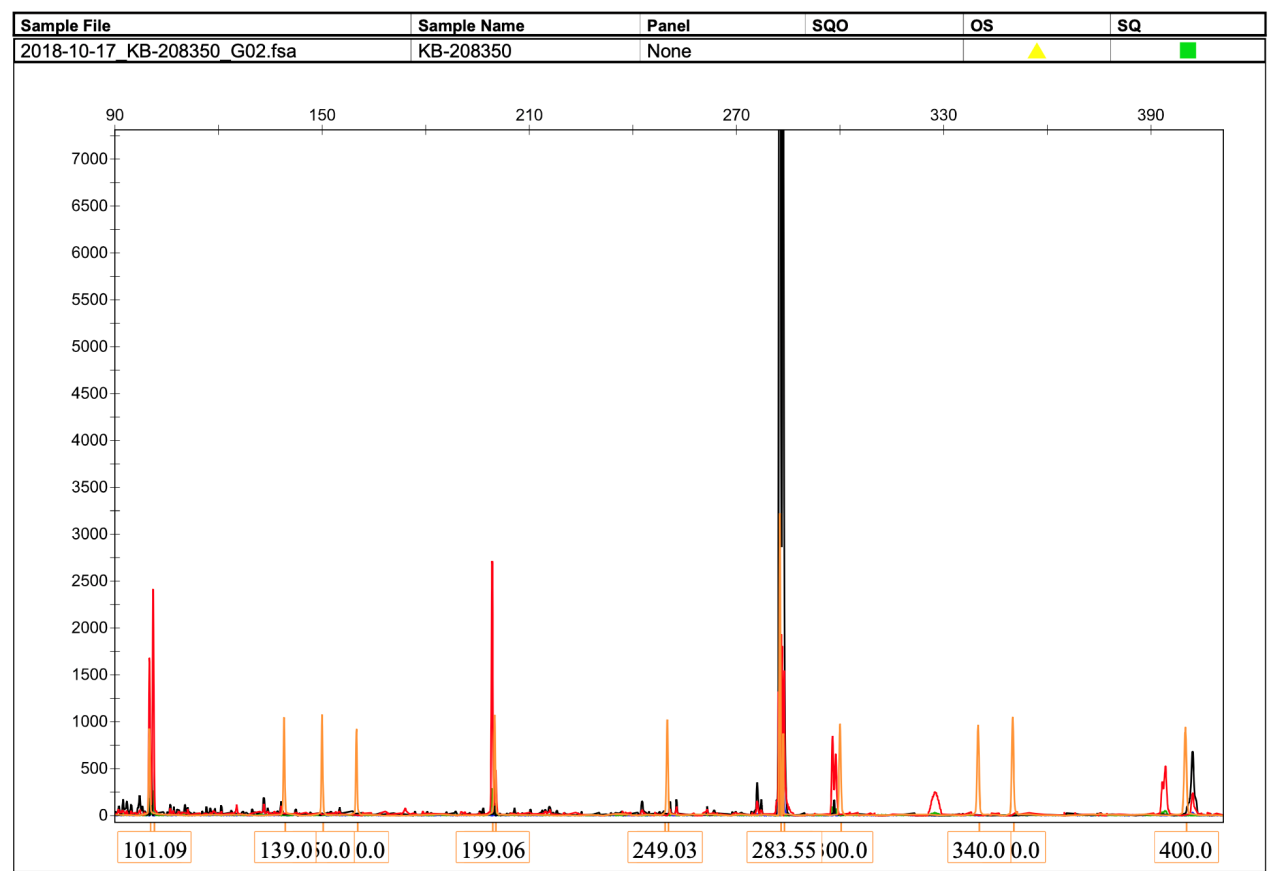


S8C
